# Two stable variants of *Burkholderia pseudomallei* strain MSHR5848 express broadly divergent *in vitro* phenotypes associated with their virulence differences

**DOI:** 10.1371/journal.pone.0171363

**Published:** 2017-02-10

**Authors:** A. A. Shea, R. C. Bernhards, C. K. Cote, C. J. Chase, J. W. Koehler, C. P. Klimko, J. T. Ladner, D. A. Rozak, M. J. Wolcott, D. P. Fetterer, S. J. Kern, G. I. Koroleva, S. P. Lovett, G. F. Palacios, R. G. Toothman, J. A. Bozue, P. L. Worsham, S. L. Welkos

**Affiliations:** 1 Diagnostic Systems Division, USAMRIID, Frederick, Maryland, United States of America; 2 Bacteriology Division, United States Army Medical Research Institute of Infectious Diseases (USAMRIID), Frederick, Maryland, United States of America; 3 Center for Genome Sciences, USAMRIID, Frederick, Maryland, United States of America; 4 Biostatistical Services Division, USAMRIID, Frederick, Maryland, United States of America; Chang Gung University, TAIWAN

## Abstract

*Burkholderia pseudomallei* (*Bp*), the agent of melioidosis, causes disease ranging from acute and rapidly fatal to protracted and chronic. *Bp* is highly infectious by aerosol, can cause severe disease with nonspecific symptoms, and is naturally resistant to multiple antibiotics. However, no vaccine exists. Unlike many *Bp* strains, which exhibit random variability in traits such as colony morphology, *Bp* strain MSHR5848 exhibited two distinct and relatively stable colony morphologies on sheep blood agar plates: a smooth, glossy, pale yellow colony and a flat, rough, white colony. Passage of the two variants, designated “Smooth” and “Rough”, under standard laboratory conditions produced cultures composed of > 99.9% of the single corresponding type; however, both could switch to the other type at different frequencies when incubated in certain nutritionally stringent or stressful growth conditions. These MSHR5848 derivatives were extensively characterized to identify variant-associated differences. Microscopic and colony morphology differences on six differential media were observed and only the Rough variant metabolized sugars in selective agar. Antimicrobial susceptibilities and lipopolysaccharide (LPS) features were characterized and phenotype microarray profiles revealed distinct metabolic and susceptibility disparities between the variants. Results using the phenotype microarray system narrowed the 1,920 substrates to a subset which differentiated the two variants. Smooth grew more rapidly *in vitro* than Rough, yet the latter exhibited a nearly 10-fold lower lethal dose for mice than Smooth. Finally, the Smooth variant was phagocytosed and replicated to a greater extent and was more cytotoxic than Rough in macrophages. In contrast, multiple locus sequence type (MLST) analysis, ribotyping, and whole genome sequence analysis demonstrated the variants’ genetic conservation; only a single consistent genetic difference between the two was identified for further study. These distinct differences shown by two variants of a *Bp* strain will be leveraged to better understand the mechanism of *Bp* phenotypic variability and to possibly identify *in vitro* markers of infection.

## Introduction

*Burkholderia pseudomallei* (*Bp*) causes melioidosis and is a Health and Human Services (HHS) Tier 1 bacterial agent. This saprophytic, free-living organism causes endemic infections in tropical regions such as Southeast Asia and Northern Australia. It is of widespread concern for reasons including its large environmental range [1], the challenges involved in disease diagnosis, treatment complications due to inherent and acquired antibiotic resistance, and its potential for adversarial use [2–7]. *Bp* is a potential biothreat agent because of its high aerosol infectivity and ability to cause severe disease with often nonspecific symptoms [2, 7].

Infections with *Bp* occur upon exposure to contaminated water, soil, or secretions, and through skin abrasions, inhalation, or ingestion. The disease is manifested by numerous and often generalized symptoms such as fever, ulcerating lesions of the skin and mucus membranes, pneumonia, abscesses in multiple organs, and septicemia. Without effective treatment, the course of melioidosis can range from acute and rapidly fatal to a protracted and chronic form; the latter being commonly associated with immunocompromising conditions such as diabetes [2, 3, 8]. Reoccurring illness is also observed and can potentially be due to reinfection or relapse of a latent infection. All of these forms, especially the more enduring ones, can be very challenging to diagnose and treat effectively [2, 3, 7].

*Bp* strain MSHR5848 was originally isolated from the sputum of a patient with suspected inhalational melioidosis. A stock of MSHR5848 maintained at the U. S. Army Medical Research Institute of Infectious Diseases (USAMRIID) and designated BURK178, was observed to produce colony variants. Strains of *Bp* typically exhibit variations in colony morphology and these variants often occur randomly and are not stably reproduced on subculture. However, BURK178 produced two distinct colony variants, designated “Smooth” and “Rough”, with numerous *in vitro* and *in vivo* phenotypic differences. Although the colony morphotypes were relatively stable under typical laboratory conditions, both Smooth and Rough could switch to the other type at frequencies which varied depending on the growth condition [9–14]. The frequent production of colony morphological variants from a single strain is a well-established bacterial phenomenon [9–22]. Morphotypic changes may be due to mechanisms such as phase variation (reversible switch between an on/off expressing phase) or to antigenic variation (expression of various alternate forms of an antigen on the bacterial surface). Either form of colony morphotype expression can result from genetic or epigenetic mechanisms which change the sequence of a gene or affect its expression without altering its sequence, respectively.

Several early studies, beginning with those reported in 1924 by Stanton et al., established that *Bp* isolates from human and animal clinical samples and from environmental sources can produce two or more colony variants [15–17]. These variants were described as rough and mucoid or smooth forms. The colony types were associated with several *in vitro* phenotypic differences and potentially with alterations in *in vivo* virulence [15, 17, 23]. Numerous recent studies have supported the hypothesis that different colony morphotypes potentially reflect adaptive changes which enhance fitness in a particular environment [12–14, 20–22].

Many studies performed by Chantratita and colleagues confirmed the predominance of variants with a rough morphology in *Bp* strains cultured from melioidosis patients [12]. This colony type (designated morphotype I) was seen on Ashdown’s medium in >75 to 93% of clinical isolates. However colony morphology varied greatly within and between samples and seven distinct colony morphologies of *Bp* were identified [12]. The rough variant was considered to be the parental type from which the others most likely arose *in vivo*. *In vitro*, the rough type could switch to the other six morphotypes in apparent response to stresses such as iron limitation [12]. These morphotypes exhibited different abilities to survive and persist in cell culture and in mice and to resist killing by peroxide and antimicrobial peptides (AMPs) [12, 13]. Similar type switching was described by Velapinato et al. for variants recovered from a patient during the acute and relapse stages of *Bp* infection [20]

Members of the *Burkholderia cepacia* complex (BCC) and the related species *Pseudomonas aeruginosa*, exhibit colony morphology switching in association with their ability to colonize the lungs and cause severe infection in patients with cystic fibrosis (CF). For example, *Burkholderia ambifaria* produces variants which differ in colony morphology, biofilm formation, plant root colonization, and virulence in a manner suggesting that *B*. *ambifaria* adapts to the very different environments of the CF lung and the rhizosphere by reversible phase variation [9].

Such bacterial-host models may provide insights useful in the analysis of phenotypic variants from *Bp* strain MSHR5848. The pathogenesis originally hypothesized for chronic lung infections involved reductive evolution in which increased nutrient availability in the airway selects for less virulent bacteria better adapted to long-term infections [24, 25]. This process involves loss of unnecessary metabolic pathways, saving energy to support persistent infection. Similarly, a case of melioidosis involving infection with chronic lung carriage led to multiple gene losses in the *Bp* lung isolates to include those encoding LPS and capsule. These changes may have reduced the host immune response to the strain, converting it to an attenuated yet persistent form. However, more recent models have proposed that evolving pathogen-host interactions produce a diverse population of phenotypically heterogeneous variants, to include ones which are fully virulent [26–29]. Thus, chronic infection and the disease environment may turn on expression of nutritional determinants and virulence factors, promoting genetic diversity and more aggressive infection [26, 30–33]. To persistently colonize, pathogens must adapt to the host and evolve with it in a way that favors its increased proliferation and ability to evade host immune responses [26, 34].

Specific phenotypic and RNA expression profiles have been linked to host-associated adaptive responses of *Bp* [12, 13, 20, 34–37],. While certain nutrient metabolism- and virulence-associated genes were often down-regulated, others were upregulated [26–29, 34, 36]. The latter included genes involved in anaerobic metabolism and energy production [20, 35, 36], resistance to stress conditions (e.g., reactive oxygen or nitrogen intermediates) [12, 20, 35, 37], and genes encoding virulence factors promoting host cell survival and spread, e.g., type six secretion system (T6SS-1) functions [20, 24]. Such genetic changes likely contribute to the increasing bacterial adaptive fitness and immune evasion mechanisms required for prolonged infection [20, 34, 35, 37]. Findings from these and other models of infection illustrate the potential role of “nutritional virulence” in pathogenesis [8, 38–40] and stimulate research on metabolism-based mitigation of disease.

The goals of this study were to phenotypically and genetically characterize the major variants of MSHR5848 and initiate efforts to determine the mechanism of variant expression and its role in disease pathogenesis. A wide ranging approach was taken utilizing multiple technologies, and it was hypothesized that an analysis of the phenotypic variation of *Bp* would help target *in vitro* markers associated with different stages of infection.

## Materials and methods

### Media and chemicals

Nonselective media used included sheep blood agar (SBA), glycerol tryptone agar (GTA) [8], brain heart infusion (BHI) agar and Luria broth (LB) agar. The four differential/selective media used included: OFPBL (oxidation-fermentation base-polymyxin B-bacitracin-lactose) agar; PC/BCA (*Pseudomonas*/*Burkholderia cepacia* agar) with polymyxin B, ticarcillin, and dye to detect alkaline pyruvate metabolism; BCSA (*Burkholderia cepacia* selective agar), with polymyxin B, gentamicin, vancomycin, sucrose and lactose with dye to detect acid production (for *Bp*); and Ashdown’s agar (AA) containing glycerol, dyes and gentamicin. All were available commercially (Thermo Fisher-Remel, Waltham, MA) except GTA and AA plates which were manually prepared as directed by the manufacturer or as described previously. Liquid growth media were LB broth, glycerol tryptone broth (GTB), or cation-adjusted Mueller-Hinton II Broth (MHB) (BBL™, BD Diagnostics Franklin Lake, NJ). Chemicals were obtained from Sigma-Aldrich (St. Louis, MO), and antimicrobial peptides were acquired from the following sources: Sigma/Fluka, Bachem (Torrance, CA), Biopeptek (Malvern, PA), Synthetic Biomolecules (San Diego, CA), and Peptides International (Louisville, KY).

### Bacterial strains and characterization

*Bp* strain MSHR5848 was originally isolated from human sputum in a suspected inhalational melioidosis case at the Royal Darwin Hospital in Australia in 2011 and was subsequently sent to the Menzies School of Health Research (MSHR) in 2012. The strain was received by the USAMRIID Department of Defense Unified Culture Collection (UCC) in 2013 and designated BURK178.

The source vial of BURK178 was propagated first into a seed stock and then the seed stock was amplified into a production lot of single use cryovials. Culturing was done using 5% SBA (Remel, Lenexa, KY) and colonies were harvested into a suspension of TSB with 12.5% glycerol. Colony morphology was initially assessed post-production on 5% SBA and AA, and cellular morphologies were assessed by performing Gram stains on each observed variant. Variants were also stained with the fluorescent DNA binding dye propidium iodide (Sigma-Aldrich). Smooth and Rough colonies were suspended in PBS and the suspensions were dried on microscope slides and stained with propidium iodide. The slides were viewed on an Olympus BX51 microscope with phase contrast (100x, oil immersion objective) and fluorescence (exe 535 nm/em 617 nm) microscopy.

#### Presence of colony variants in strain stocks

Purity and colony morphology assessments were performed on the original source vial as well as the seed and production stock preparations. A ten-fold serial dilution of each stock was prepared using Dulbecco’s Phosphate Buffered Saline (DPBS) as the diluent. In order to obtain single colonies in quantities between 30 and 300 per plate, 100 μl of the 10^−6^ and 10^−7^ dilutions were used. The original stock was slightly less concentrated so 50 μl the 10^−5^ and 100 μl of the10^-6^ dilutions were used instead. Both 5% SBA and AA were inoculated with the respective dilutions using a cell spreader and an inoculating turntable. In all cases, each dilution was plated in triplicate. The plates were incubated at 35°C in ambient atmosphere for a total of 96 h with observations occurring approximately every 24 h. In addition to standard colony morphology assessments, each plate was also photographed and the colonies were counted during each observation period.

To characterize the morphological stability of each variant, a subsequent dilution series of each variant was prepared using a single representative colony as the starting material. The dilution series was prepared by suspending the single colony in 1 ml DPBS and conducting another ten-fold serial dilution. One hundred microliters of the 10^−6^ and 10^−7^ dilutions were used to inoculate 5% SBA plates in triplicate. Plates were incubated and assessed as described above. Each observed variant was characterized by all of the phenotypic and molecular methods detailed below.

### *In vitro* growth and variant switching frequency

Growth rates in broth culture of MSHR5848 Smooth and Rough were assessed using the BioScreen C automated growth curve analysis system by Growth Curves USA. Each variant was first propagated on 5% SBA at 35°C for 48 h in ambient atmosphere. A single representative colony from each culture was then chosen to inoculate an MHB starter culture, which was subsequently distributed to the wells of a BioScreen C plate. The starter culture was incubated on the BioScreen C at 37°C with continuous shaking and data were collected every 15 min at 600nm. When the starter culture reached an OD_600_ of 0.1–0.3, the starter culture was removed from the instrument and transitioned to a primary culture. The following formula was used to determine the volume of starter culture (V_s_) to add to fresh media with a volume (V_m_)_,_ depending on the starter OD (OD_s_): V_s_ = (0.001/OD_s_)*V_m_. The new suspension was vortexed and distributed to the wells of a fresh BioScreen C plate. The primary culture was incubated for 48 h in the same manner as the starter culture. Variant growth curves were analyzed by two-way ANOVA on data collected at 15 minute intervals of the Bioscreen C program run. At each time point a two-way ANOVA was performed, with the two factors being the colony type and the stock (original source or production). The interaction between the two factors was not significant, indicating that the effect of colony type was homogenous across stocks. Following removal of the interaction term, the statistical significance of the main effect of colony type from the two-way ANOVA was obtained. The doubling times of Smooth and Rough variants were determined by curve fitting and analysis implemented in SAS Proc NLIN. The latter is a procedure for fitting nonlinear models in SAS statistical software version 9.4, based on the slopes of the curves at their inflection points. The doubling times were estimated by OD_t_ = OD_0_(2)^t/d^ where OD_t_ is the number of bacteria (OD) after time (t), OD_0_ is the number of bacteria at t_0_, and d is the doubling time. The statistical significance of the two doubling time values was determined by Welch’s t-test.

Multiple stocks ("clones") were prepared of individual Smooth and Rough colonies from the MSHR5848 stock: 21 Smooth and 17 Rough. These clones were tested for transition to the other type in the 14 growth conditions described below. The circumstances tested in 1–10 corresponded to those detailed by Wikraiphat et al. [22], 11 was described by Austin et al. [21], 12 and 14 were developed in this study, and 13 was performed according to the conditions depicted by Butt et al. [41]. Six of the Smooth clones tested had been fresh revertants of Rough clone #1 that had been incubated in condition 9 (7 days at 37°C).

### Identification and phenotyping assays

All cultures used in phenotypic tests except as indicated were propagated on 5% SBA and incubated for 48 h in ambient atmosphere at the indicated temperatures.

Fatty acid analysis was performed using the MIDI, Inc. microbial identification system (MIS) in conjunction with the Sherlock^®^ software. For MIS characterization, sample extracts were prepared in accordance with the Sherlock^®^ Microbial Identification System Operating Manual (MIDI, Inc., Newark, DE). Sample extracts were then injected into an Agilent 6850 gas chromatography system (Santa Clara, CA) where fatty acid methyl esters are separated as they pass through a capillary column and are subsequently burned by the flame ionization detector to create a signal. The data are plotted on a chromatogram and analyzed by the Sherlock^®^ Microbial Identification System software based on the retention time, size and shape of each peak. Peak profiles for each variant were searched against the Sherlock^®^ bio-threat library for species identification.

Samples were analyzed for species identification on the VITEK^®^ 2 Compact using the Gram-negative identification card (GN). The GN Card contains 47 substrates and 1 negative control and measures carbon source utilization, enzymatic activity and resistance. Suspensions of the test samples for VITEK^®^ 2 Compact assays were prepared by transferring several isolated colonies from the agar plate to 3.0 ml of 0.45% saline and adjusting the turbidity as necessary until a 0.5–0.63 McFarland standard was achieved using the DensiCHEK^TM^ Plus Meter, in accordance with the VITEK^®^ 2 Systems Product Information (bioMerieux Inc., Hazelwood, MO). Suspensions were then used to inoculate VITEK^®^ 2 GN cards. Inoculation of cards, incubation, and analysis were done automatically by the VITEK^®^ 2 Compact instrument.

The GEN III MicroPlate™ system (GEN III OmniLog^®^) was used for species identification and abridged phenotypic profiling. These plates contain 71 carbon sources and 23 antimicrobial chemicals. Inocula for all samples were prepared with inoculating fluid IF-A using protocol A in accordance with the OmniLog Data Collection Software Identification System User Guide, version 2.1 (Biolog, Inc., Hayward, CA). Data were collected every 15 min using the full data logger option for 36 h. Samples remained in the instrument beyond the completion of the original 36 h incubation period and a single data point was collected at the 48 h time point. Sample identifications were produced using a combination of the GEN III database and manufacturer assisted user database. The metabolic profiles were also evaluated by exporting the threshold values using the Biolog Retrospect software, version 2.1.1. The arbitrary respiratory units are normalized on a 0–100 scale by an algorithm of the Biolog program, which also produces threshold values. These were used to establish positive and positive/negative cut-off values, and allowed differences in metabolism by the bacteria to be discriminated for the chemicals.

#### Biolog Phenotype Microarray^TM^ (PM)

The PM system of Biolog consists of 20 microplates and includes a total of 1,920 substrates/chemicals. PM plates 1–20 are used to characterize strains in their ability to use different compounds as sources of carbon, nitrogen, phosphorus and sulfur; or in their sensitivity to stressful environmental conditions such as pH extremes or high salt concentrations; and antimicrobial chemicals such as antibiotics, detergents, oxidizing agents, and others. Sets of PM plates 1–20 for each variant were prepared in accordance with the Biolog PM procedure for *E*. *coli* and other gram–negative bacteria. Kinetic data were collected every 15 min at 37°C for 48 h and subsequently analyzed using version 1.20.02 of the File Management/Kinetic Plot and Parametric software.

The data generated from the 20 PM plates were evaluated statistically to compare target responses of the MSHR5848 morphotypes and to rank those substrates producing the greatest differences in responses between the Smooth and Rough variants. The area under the curve (AUC) parameter was selected, and four different statistical methods were used to analyze the data: the Biolog Phenotype Microarray^TM^ analysis software; the opm and Pipeline packages based on R language, as described previously [42–44]; the DuctApe suite [45]; and a two-way ANOVA model using data normalized to positive control scores, as implemented in SAS® system's PROC GENMOD procedure. The results of these analyses were in general agreement on the trends suggested by a comparison of the variants’ responses. The analysis using the PM version 1.20.02 of the File Management/Kinetic Plot and Parametric software are presented. For an analysis using the PM software, the Smooth variant served as the test and the Rough variant served as the reference. Since there was a notable signal in the A1 wells of plates 1–8, the A1 zero function was employed. The difference in the AUC between the two variants was analyzed to identify the substrates that elicited the greatest difference in response. In instances where the Rough variant had a greater response than the Smooth variant, a threshold of -3,000 omnilog units (OU) was used to select the most discriminating substrates. Due to an overall increased response of the Smooth variant, two separate thresholds were used to discriminate the substrates for which the Smooth variant had a greater response: 25,000 OU for the metabolic pathway assays and 11,000 OU for the chemical sensitivity assays. These thresholds generated lists of a reasonable size to use in further exploration of functional significance.

#### LPS profiling

*Bp* MSHR5848 Smooth and Rough variants were streaked onto 5% SBA plates and incubated at 37°C for 48 h. From each plate, approximately 5–6 isolated colonies with uniform morphology (i.e., all Smooth or Rough) were resuspended in GTB broth and incubated at 37°C with shaking at 200 rpm overnight. The cultures were then diluted to an OD_620_ of approximately 1.0 and heat-killed at 90°C for 90 min. After sterility was confirmed, LPS was extracted using the procedure from Yi and Hackett [46]. The purified LPS samples were separated by SDS-PAGE using 10–20% Tricine gels (Thermo Fisher Scientific), and western blots were performed using a variety of monoclonal antibodies specific for *Burkholderia* LPS. These included 11G3-1 from Dr. D. Waag and S. Trevino; BP7 1H7, BML 11G6, BPL 30D11, BP7 2G6, BML 18F8, and BP A2 from Dr. S-C. Lo; 9D5 from Dr. N. Chantratita; and 3D11 from LPBio, Inc. Peroxidase-labeled goat anti-mouse IgG was used as the secondary antibody (KPL, Gaithersburg, MD), and the blots were developed using colorimetric detection with tetramethylbenzidine (TMB) Membrane Peroxidase Substrate (KPL). Alternatively, silver staining was conducted using a method described by Tsai and Frasch [47].

#### Antimicrobial sensitivity testing

Chemical and antimicrobial peptide sensitivity testing was performed using the GEN III MicroPlates™, described above, and additional microtiter tests were used to evaluate sensitivity to selected chemicals and antimicrobial peptides (AMPs). Chemicals selected for testing in the microtiter assays included NaCl (1%, 4%, and 8%), nalidixic acid (5 and 50 μg/ml), the surfactant niaproof 4 (0.027% and 0.10%), reactive oxygen species (ROS) inducer paraquat dichloride (2.5 μM), and reactive N_2_ intermediate (RNI) sodium nitrite (2 mM). *Bp* strains were differentially responsive to the selected concentrations in preliminary tests; and all were sensitive to 2.5 μM paraquat and to 2 mM sodium nitrite as reported [13]. Eight AMPs were screened: cecropin A, mastoparan 7, LL-37, magainin, melittin, BMAP-18, bactenecin, and CA-MA [13, 48–50]. *E*. *coli* strain ATCC 25922 and *Bp* K96243 were used in the assays to verify activity. After addition of the antimicrobials to the trays, the wells were inoculated with strains adjusted to a concentration of 1 x 10^6^ CFU/ml in MHB. The trays were incubated for 48 h at 37°C, and growth or inhibition in all assays was determined by reading absorbance (A_630_). The absorbance results were recorded as resistant (R, OD_630_ >75% positive growth control), sensitive (S, OD_630_ <50% positive control), borderline (R/S, OD_630_ ≥50 and <75% positive control), or very sensitive (S+, OD_630_ ≤2x the uninoculated negative control wells containing medium alone). Differences in absorbance values determined from replicate GEN III plate experiments were evaluated by t-test or by ANOVA and Tukey multi-comparison post-tests, as needed. These statistical analyses were performed with GraphPad Prism ver. 5.2.

#### Persistence phenotype

An *in vitro* assay to detect persister-type cells was performed to differentiate cells based on tolerance to high concentrations of antibiotic. The procedure used was described previously by Butt and coworkers [41] who identified the HicAB system as having a role in persistence in an *in vitro* antibiotic tolerance assay. To test the Smooth and Rough variants for possible differences in persister cell formation, an *in vitro* assay comparing their development of tolerance to a high concentration of ciprofloxacin was employed, as described previously [41].

For both variants, the growth of log and stationary phase cultures in LB with ciprofloxacin added (391 μg/ml, 100x MIC) were compared by dilution plating at intervals during incubation at 37°C for 30 h, as described previously [41].

### Molecular genetics

Ribotyping was performed on variants which had been subcultured on 5% SBA and incubated for 48 h at 35°C in ambient atmosphere. Cultures of each sample were digested with EcoRI according to the Dupont Qualicon Riboprinter^®^ Microbial Characterization System User’s Guide (Dupont Qualicon, Wilmington, DE). The patterns were analyzed using BioNumerics version 7.5 (Applied Maths, Austin, TX).

For MLST analysis, DNA was extracted from single colonies for each observed variant using heat lysis. PCR amplification and sequencing of seven housekeeping gene fragments was subsequently performed using primers for the *Bp* MLST scheme, as previously described by Godoy, et al. [51].

#### Whole Genome Sequence (WGS) analysis

To identify genetic changes associated with the two phenotypic variants, we conducted whole genome sequencing. Two preparations were characterized for both MSHR5848 Smooth and Rough variants. In the first, the strains were propagated in GTB [8] starting from a single representative colony for each variant and incubated with shaking to log phase. The bacteria were harvested and genomic DNA was extracted using the Qiagen DNeasy Blood and Tissue kit and sequenced on both a Pacific Biosciences (PacBio) RSII and an Illumina MiSeq. For the second preparation, DNA was extracted directly from representative colonies without additional growth in broth culture, thus explicitly controlling for potential reversion events. For each variant, approximately five colonies were pooled and DNA was extracted with the Qiagen EZ1 Biorobot using the Virus 2.0 kit and sequenced on an Illumina MiSeq.

PacBio libraries were prepared using the SMRTbell™ Template Prep Kit (Pacific Biosciences, Menlo Park, CA) following manufacturer’s protocol. DNA (5 μg) was fragmented using gTUBE (Covaris Inc., Woburn, MA) to ~20 kb. After DNA damage repair and ends repair, blunt hairpin adapters were ligated to the template, and failed ligation products were digested with ExoIII and ExoVII exonucleases. Resulting SMRTbell template was size selected on BluePippin system (Sage Science, Beverly, MA) using 0.75% dye-free agarose cassette with 4-10kb Hi-Pass protocol and low cut set on 4 kb. Size selected template was cleaned and concentrated with AMPure PB beads. The P4 polymerase was used in combination with the C2 sequencing kit and we collected 180-minute movies. Raw reads were quality filtered (subread length > = 500; polymerase read quality > = 0.80) and *de novo* assembled using HGAP 3 v2.2.0. Assemblies were checked for redundant sequence at the ends using Gepard v1.3.For each complete circular chromosome, these redundant sequences were trimmed to one copy and a new breakpoint was chosen for its linear representation. Reads were then re-aligned to the trimmed and shifted draft assembly for correction using the Quiver algorithm. The final genome assemblies (GenBank: CP016909—CP016912) were annotated using NCBI’s Prokaryotic Genome Annotation Pipeline v3.2 [52].

Illumina libraries were prepared using the Nextera XT kit according to manufacturer’s instructions and were sequenced with a V2 500 cycle kit or a 300 cycle paired end sequencing kit. Reads were quality filtered using Prinseq-lite v0.20.3 and Illumina adaptors were clipped using Cutadapt v1.2. Reads were then mapped using Bowtie2 v2.0.6 against the NCBI RefSeq MSHR5848 chromosomal sequences (GenBank: NZ_CP008909—NZ_CP008910). Samtools v1.2 was used to remove 1) reads that mapped equally well to multiple places in the reference genome and 2) reads that did not map in a proper pair. PCR duplicates were removed with Picard v1.131. Variants were called using the UnifiedGenotyper in GATK v3.1-1-g07a4bf8 and the predicted effects of variants were annotated with SnpEff using a database prepared from the MSHR5848 reference GFF downloaded from GenBank.

### Macrophage infection assays

Phagocytosis assays to measure the ability of the MSHR5848 variants to infect macrophages and to induce cell damage were performed as described in detail previously [8]. In brief, J774.A1 mouse macrophages in a 24-well tray were infected with 10–20 CFU *Bp* and incubated 1 h to allow phagocytosis of the bacteria. Samples of lysed cells were collected for viable counts (“1 h”), or the infected wells were incubated for 2 h in the presence of 250–500 μg/ml kanamycin to kill unphagocytosed bacteria. Lysed samples were collected (“3 h”) and the plate incubated an additional 5 h (“8 h”) at which time lysed wells were sampled for viable counts. In addition, in separate wells, the extent of cytotoxicity and of cell loss was measured by trypan blue (TB) dye uptake and/or by staining with propidium iodide (PI). Live cells exclude TB and PI and are unstained under phase (TB) or fluorescence (PI), whereas dead cells are permeable and have blue (TB) or bright red (PI) stained nuclei. Cells on coverslips were alternately stained with Diff-Quik™ histologic stain to assess macrophage condition (normal versus necrotic, apoptotic, or multinucleated appearance of cells and nuclei), extent of formation of multi-nucleated giant cells (MNGC), and the relative level of residual bacterial infection. Differences in the viable counts obtained from infected macrophages were evaluated by t-test or by ANOVA and Tukey multi-comparison post-tests, as needed. These statistical analyses were done with GraphPad Prism ver. 5.2.

### Mouse infection

BALB/c mice were challenged by the intraperitoneal (IP) route with *Bp* MSHR5848 strain, Smooth or Rough, and the mice were monitored for morbidity and mortality for 60 days, as described previously [8]. Survival data from days 21 and 60 were evaluated statistically by Bayesian probit analysis to calculate median lethal dose (LD_50_ and 95% credible estimates) and determine dose response associations, as described in detail previously [8] and in the text. Bayesian lethal dose estimates and significance were determined using Stan statistical software version 2.1.0 and other lethality rate statistics were performed using the statistical software R version 3.1.1.

### Ethics statement

Animal research at the United States Army Medical Research Institute of Infectious Diseases (USAMRIID) was conducted under an animal use protocol approved by the USAMRIID Institutional Animal Care and Use Committee (IACUC) in compliance with the Animal Welfare Act, PHS Policy, and other Federal statutes and regulations relating to animals and experiments involving animals. The facility where this research was conducted is accredited by the Association for Assessment and Accreditation of Laboratory Animal Care International (AAALAC) and adheres to principles stated in the Guide for the Care and Use of Laboratory Animals (National Research Council, 2011). Challenged mice were observed at least daily for 60 days for clinical signs of illness. Humane endpoints were used during all studies, and mice were humanely euthanized when moribund, according to an endpoint score sheet. Animals were scored on a scale of 0–11: 0–2 = no significant clinical signs (e.g., slightly ruffled fur); 3–7 = significant clinical symptoms such as subdued behavior, hunched appearance, absence of grooming, hind limb issues of varying severity and/or pyogranulomatous swelling of varying severity (increased monitoring was warranted); 8–11 = distress. Those animals receiving a score of 8–11 were humanely euthanized. However, even with multiple observations per day, some animals died as a direct result of the infection.

## Results

### Morphological characterization

#### Colony and microscopic characterizations

**Fig 1:**
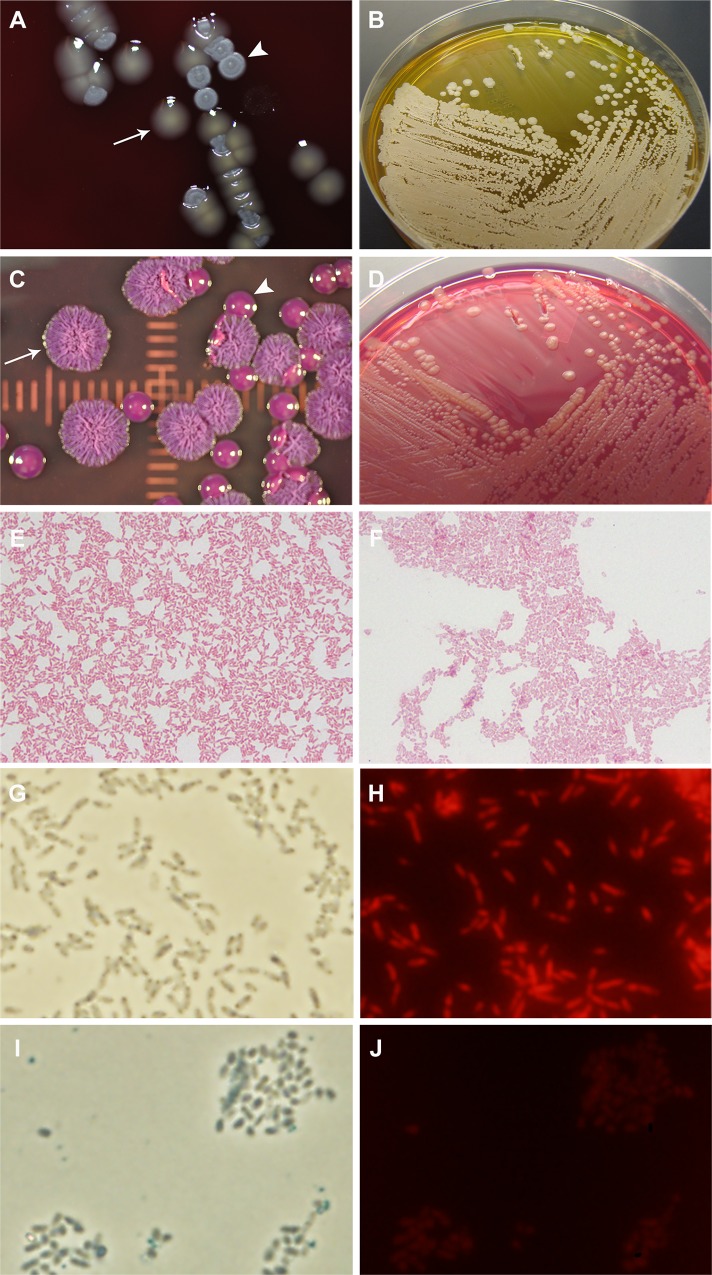
Smooth and Rough variant colony and microscopic morphologies. A: Sheep blood agar plates. The smooth, glossy, pale yellow colonies are the Smooth morphotype (arrow) and the flat dry grey-white colonies are Rough (arrowhead). B: Ashdown’s agar plates. The raised shiny, smooth deep pink colonies are Smooth (arrow) and the wrinkled dry pink and purple colonies are Rough (arrowhead). C and D: BSCA plates with Rough (lactose-positive, yellow dye) and Smooth (lactose-negative, red dye) growth, respectively. E and F: Gram stains of Smooth and Rough, respectively (100x objective). The Rough cells exhibit a”safety pin” appearance with stained poles and unstained center whereas the Smooth bacteria have a typical gram-negative rod morphology. G–J: The BURK production stock was grown on SBA plates at 37°C for three days. Smooth or Rough colonies were suspended in PBS and the suspensions were dried on microscope slides and stained with the fluorescent DNA binding dye PI. The slides were viewed on an Olympus BX51 microscope with phase contrast (100x, oil immersion objective) or fluorescence (ex. 535 nm/em. 617 nm) microscopy. G: Smooth colony bacteria viewed by phase contrast. H: Smooth colony bacteria stained with PI and viewed with fluorescence. I: Rough colony bacteria viewed by phase contrast. J: Rough colony bacteria stained with PI and viewed with fluorescence.

BURK178 exhibited two distinct colony types when cultured on various differential and selective plated media (Table 1). On 5% SBA plates, BURK178 produced a smooth pale yellow colony (Smooth) and a flat rough and greyish-white colony (Rough). Fig 1A displays representative Smooth and Rough colonies on 5% SBA. On AA, the Rough variant exhibited colonies resembling the morphotype I colony morphology described by Chantratita, et al. [12], while the Smooth variant primarily yielded colonies consistent with the morphotype III or VI morphology, shown in Fig 1B. BCSA plates are commonly used for selective isolation of pathogenic *B*. *cepacia* complex species and for *Bp*. In addition to antibiotics for selection, the medium contains lactose and a pH indicator to detect strains capable of lactose utilization with the production of acid. As shown in Fig 1C and 1D, the MSHR5848 Rough variant appeared to ferment lactose and produce acidic conditions on BSCA as detected by the change in color to a greenish-yellow. In contrast, MSHR5848 Smooth colonies were lactose-negative and produced a pink color change attributed to alkaline metabolism of peptones.

**Table 1:** Summary of phenotypic differences of *Bp* MSHR5848 (BURK178) variants.

Phenotype		Difference	Smooth^a^	Rough^b^
Morphologic	Gram stain	Yes	GNR	typical *Bp*
	PI nucleic acid dye	Yes	positive	negative
	Colony morphology	Yes	raised, yellowish, shiny	flat, grey, dry
	BCSA Sugar utilization	Yes	alkaline	acid
Biochemical	BiOLOG GENIII	Yes^c^	*Bp* (36 h)	4 *Burkholderia* spp. (36 h)
	BiOLOG GENIII	Yes^c^	*B*. *thailandensis* (48 h)	*Bp* (48 h)
	VITEK2™	No^c^	*Bp*	*Bp*
	SherlockMIDI	No^c^	*Bp*	*Bp*
	Antimicrobial sensitivities	Yes^d^	variable	variable
Phenotype Analyses	GENIII microarray^e^	Yes	15/15 positive	7/15 positive
	PM metabolic activity^f^	Yes	more active (59 significant)	less active (47 significant)
Molecular Analyses	DNA sequences	Yes	WGS difference (chrom 2)^g^	3-base deletion
	MLST^h^	No	-	-
	Riboprinting	No	-	-
Infection	Macrophage cytotoxicity^i^	Yes	75% killing	12.5% killing
	Macrophage replication^i^	Yes	≥ fivefold greater uptake	reduced
	Mouse virulence (IP)	Yes	less virulent	more virulent^j^
Other	LPS banding pattern	No	type A subtype 3	Same
	Persister phenotype^k^	No	positive	Same

^a^The Smooth morphotype from the solid medium production stock of strain MSHR5848.

^b^Phenotypes of the Rough variant of the MSHR5848 solid medium production stock are similar to those of the liquid medium production stock of Rough.

^c^The species identifications of the variants reported in the GENIII system differed at the 36 h and 48 h readings. VITEK2™ and SherlockMIDI identification systems reported no differences; all variants were *B*. *pseudomallei*.

^d^Inter-experimental variation in sensitivities of the Smooth and Rough strains to selected chemicals and antibiotics in manual microtiter assays.

^e^Based on GENIII plates (94-phenotypes) incubated and analyzed on the Omnilog.™ Of the 15 carbon source substrates differing most consistently between the variants, 15 were strongly metabolized by Smooth and 7 were strongly metabolized by Rough.

^f^Metabolic activity of variants for 1,920 substrates or inhibitors (20 PM plates) measured during incubation in Biolog Phenotypic Microarray™.

^g^Sequences from WGS libraries of variant Smooth and Rough colonies were obtained using the PacBio and Illumina MiSeq platforms. A 3-base sequence on chromosome 2 of Smooth was absent in Rough and may impact the downstream gene as described in the text.

^h^The multilocus sequence type (MLST) was no. 553 for both variants; their Ribotyping Riboprinter™ rRNA patterns were conserved.

^i^Smooth variant was more cytotoxic than rough in terms of cell killing and MNGC production; and was phagocytosed to a fivefold greater extent and replicated significantly more as detailed in Fig 5 and Table 5.

^j^LD50 values (days 21 and 60) of MSHR5848 Rough were almost tenfold lower (and had higher dose-related lethality rates) than MSHR5848/Smooth. Fig 6 confirms the more rapid loss of survival of Rough.

^k^This phenotype is based on an in vitro model for development of tolerance to high levels of ciprofloxacin. Both strains exhibited persister tolerance.

In addition to being visually distinct on blood agar, the variants also differed at the microscopic level (Table 1). Gram stains showed that the Rough variant has the typical safety pin appearance associated with *Bp* (data not shown), while that characteristic is not as obvious in the Smooth variant (Fig 1E and 1F). Also, Smooth, but not Rough, was stained with DNA-binding dye propidium iodide (Fig 1G–1J), a finding which supports previous observations that certain mucoid isolates of *Bp* secrete DNA [21].

#### Analysis of colony variants in stock cultures

Stock cultures were prepared from the BURK178 source vial for experimental use. Seed and production stocks prepared on 5% SBA produced both Smooth and Rough colonies. The percentage of Smooth colonies increased from 0.35% in the source vial to 4.7% in the seed stock, and increased again to levels nearly equal (47%) to those of the Rough variant in the production stock. Thus, the ratio of Smooth to Rough increased with each subsequent stock preparation (S1 Table) and appears to be related to growth rate differences as described below.

Representative colony types isolated from the seed and production stocks were collected, serially diluted, and plated for single colony isolation to characterize the morphotypes they in turn produced. All colonies observed in platings of individual Rough colonies from the BURK178 seed or production stocks exhibited the Rough morphology (S2 Table). Similarly, except for the production of low numbers (0–2.6%) of random unstable variants (mucoid or flat, smooth grey morphotypes in S2 Table), 92–95% of the colonies produced by the Smooth variant were also Smooth. In contrast, both of the random unstable variants produced by the Smooth colony yielded colonies the majority of which displayed typical Smooth morphology and not the morphology of the unstable variant (S2 Table and data not shown). This is illustrated by the results with the “mucoid” variant from the seed and production stocks, which produced Smooth colonies present at 79% to 91% of the total colony count (S2 Table).

### Overview of phenotypic differences

To analyze the phenotypic and genotypic properties affected by the variant switching and begin to understand its mechanism, a wide range of characteristics were compared for the two major variants. These characteristics and assays performed are listed in Table 1 and the Smooth and Rough responses summarized. As illustrated in Table 1, many different phenotypes were affected by the switching process, ranging from *in vitro* metabolic activity to virulence for mice.

#### *In vitro* growth and variant switching frequency

The Smooth variant multiplied at a higher rate *in vitro* and reached stationary phase before the Rough variant. The Smooth and Rough growth curves were analyzed by two-way ANOVA on data collected every 15 min during the automated Bioscreen C run. For all time points, Smooth and Rough growth differed significantly (P values from 0.0037–0.0001). The doubling times of the variants were 1.74 ± 0.1 h and 2.08 ± 0.21 h for Smooth and Rough, respectively; they were statistically different (P = 0.0001).

The frequency of switching between the two major colony variants present in the MSHR5848 stock was examined by identifying conditions under which the Smooth and Rough variants of MSHR5848 could revert or switch to the other morphotype. None of the Smooth single colony stocks that were tested directly exhibited switching to the Rough morphotype (Table 2). The six Smooth clones for which switching was observed had been freshly-isolated revertants of a Rough colony stock (Table 2). These six Smooth isolates were tested for reversion to Rough using condition 14 (shaking incubation for 24h at 37°C in TSB) or direct plating of the frozen stocks onto TSA and 5% SBA with incubation for 3days at 37°C. Only condition 14 induced reversion of Smooth to Rough, with a mean frequency of 2.1% (Table 2, and data not shown). The Rough variant overall reverted more frequently and in more growth conditions than was observed for Smooth, although the switching frequency was variable between experiments (Table 2, and data not shown). Reversion of Rough colonies to Smooth was not observed for six conditions, occurred with low frequency in conditions 2, 3, 6, and 7 (from 0.1 to 1.6% of colonies plated), and occurred more frequently in conditions 4 and 5 (up to 5.1% and 2.3%, during growth in high pH conditions or at 42°C, respectively). For the latter, the frequency of Rough to Smooth switching increased to 30.5% when the cultures were incubated for 6 days (42°C). Reversion was detected most often with condition 9 (mean of 20.9%), but was variable between experiments in both the number of Rough single colony strains that produced revertants and the percentage of Smooth colonies produced. Condition 9 involved incubation in TSB without shaking at 37°C for 7 days.

**Table 2:** Growth conditions used to produce colony morphotype switching in MSHR5848 variant colony stocks^a^.

6				Incubation			Colony reversion^b^	
No.	Medium	Addition	Temp	Atmosphere	Shaking	Time and sampling	Smooth^c^	Rough^c^
1	Distilled water	-	37°C	aerobic	no	24 h	0/11	0/11
2	TSB, pH 7.4	-	37°C	aerobic	no	24 h	"	2-Feb
3	TSB, pH 4.0	-	37°C	aerobic	no	24 h	"	2-Feb
4	TSB, pH 8.5	-	37°C	aerobic	no	24 h	"	2-Feb
5	TSB, pH 7.4	-	42°C	aerobic	no	24 h	"	2-Feb
6	TSB, pH 7.4	350mM NaCl	37°C	aerobic	no	24 h	"	1-Jan
7	TSB, pH 7.4	50mM NaNO2	37°C	aerobic	no	24 h	"	1-Jan
8	TSB, pH 7.4	2mM H_2_O_2_	37°C	aerobic	no	24 h	"	0/11
9	TSB, pH 7.4	-	37°C	aerobic	no	7 days	"	4-Apr
10	TSB, pH 7.4	-	37°C	hypoxic^d^	no	24 h	"	0/11
11	LB	-	37°C	aerobic	yes	serial plating, 2–72 h^e^	"	0/11
12	SBAP	-	37°C	aerobic	no	3 days	"	0/11
13	LB	ciprofloxacin	37°C	aerobic	no	stationary phase culture^f^	"	0/11
14	TSB, pH 7.4	-	37°C	aerobic	yes	24 h^g^	6/6^h^	nd

^a^Single colony stocks of each variant were prepared, 21 of Smooth and 17 of Rough. Growth conditions tested for reversion (#1–14) are described in the Materials and Methods.

^b^Number of single colony stocks with revertants to the other type for the total number stocks tested. The mean frequency of reversion to the other type for each condition ranged from 0.8 to 21% (range of 1–75% in individual tests).

^c^A total of 17/21 Smooth colony stocks were tested in conditions #1–12 and 14; and a maximum of 13/17 Rough colony stocks were tested in conditions #1–13. For conditions 2, 3, and 5–7, the one to two stocks of Rough tested switched to Smooth at very low rates (0.1 to 1.3%).

^d^Incubated in GasPak jar with an anaerobic gas-generating system.

^e^As described by C. Austin et al., 2015

^f^Serial plating (0 h–30 h) of stationary phase culture, as described by A. Butt et al., Biochem. J., 2014

^g^Performed with freshly isolated Smooth revertant from Rough colony stock 1, after the latter had been incubated 7 days at 37°C (condition #9).

^h^The six Smooth revertants produced Rough variants in condition #14.

### Species identification

Three platforms were used for comparative species identification results for the MSHR5848 variants. In the Biolog GEN III MicroPlate^TM^ system, the Smooth variant was correctly identified as *Bp* in two out of three runs at the 36 h time point but was mis-identified as *B*. *thailandensis* in all three runs at the 48 h time point. Conversely, the identification of the Rough variant varied among four different species of *Burkholderia* at the 36 h time point but was identified correctly as *Bp* in all three runs at the 48 h time point (Table 1, and data not shown). Since GENIII identifications are based on carbon source utilization and chemical sensitivities, these ambiguous results suggested that Smooth and Rough differed significantly in their metabolic characteristics. In general, Smooth was more metabolically active than the Rough variant, an observation consistent with both the GEN III and Biolog PM phenotyping result (described below).

The Sherlock MIS and VITEK^®^ 2 systems correctly identified each variant as *Bp* with high confidence calls, according to the manufacturer’s classification. However, on both platforms, the raw data showed differences between the two variants similar to what was noted in the Biolog experiments. In the VITEK^®^ 2 system, seven substrates were utilized by the Smooth variant but not used by the Rough variant (D-mannose, D-cellobiose, malonate, D-sorbitol, citrate, D-maltose, and coumarate) (data not shown). No other differences were observed between the three strains. Although both MSHR5848 variants were identified correctly as *Bp* by the Sherlock MIS system, there were notable peak profile differences between the two variant types (Supplementary Information S3 Table). In some cases, the Smooth variant possessed peaks that the Rough variant did not, such as the 15:1 w6C and 17:1 w7c peaks. In other cases, the opposite was true. For example, the profile for the Rough variant included the 18:1 w9C, 17:0 iso 3OH and the 19:1 w6w/w7c/19cy peaks while the Smooth variant did not. In some instances, while both colony types possessed a particular peak, such as the 17:0 cyclo and the 18:1 w7c peaks, the percent contribution of that peak to the overall profile varied considerably. There were also several peaks that were present in the same proportion in both variant profiles, such as the 12:0, 13:1 and 14:0 peaks.

### Antimicrobial sensitivity testing and biochemical phenotyping

#### Biolog GENIII microplates

The sensitivities to antimicrobial chemicals were initially tested in assays with Biolog GEN III microplates, which contain 23 antimicrobial chemicals. In addition, five chemicals and eight AMPs were evaluated separately in microtiter plate assays. Inter-experimental variability in *Bp* responses of the Smooth and Rough variants was observed, as described generally for *Bp* strains [12, 13, 21, 22]. The only relatively consistent differences between Smooth and Rough were in sensitivity to the toxic amino acid D-serine and the cationic AMP magainin II [53–55]. Smooth was generally more sensitive than Rough in GENIII tests with D-serine but responses to this chemical did not significantly discriminate the variants in the latter phenotype microarray studies. Whereas Smooth was sensitive to borderline in 3/6 assays with magainin II, Rough was resistant (5/6) or borderline (1/6) to the AMP (data not shown).

The GENIII microplates additionally contain 71 carbon sources. The Smooth variant was generally more metabolically active than the Rough variant. A total of 32 substrates differed between the Smooth and Rough variants in at least one of three GENIII tests; and the 15 substrates which consistently differed between strains in all three tests are shown in Table 3. It was readily apparent that the Smooth variant metabolized a wider range of carbon sources compared to the Rough variant.

**Table 3:** Metabolic profiling of MSHR5848 variants using the 94 GENIII phenotypes^a^.

	Mean threshold values^b^	
Phenotype	Smooth	Rough
N-Acetyl-D-Glucosamine	93	33.3
N-Acetyl-D-Galactosamine	97	30
D-Mannose	93	71.7
D-Sorbitol	90.7	57
D-Mannitol	93	47.7
Myo-Inositol	96	59.3
Glycerol	95	16
Glycyl-L-Proline	47.7	8.3
L-Arginine	96	4.7
L-Aspartic Acid	92.7	17
L-Pyroglutamic Acid	96.3	8.7
L-Serine	94	41.7
D-Gluconic Acid	95.3	54.3
Citric Acid^c^	91.7	21.7
α-Keto-Butyric Acid	94	85.7

^a^A total of 32 substrates (of 71 carbon sources and 23 antimicrobial chemicals) differed between the two strains in at least 1 of 3 tests done. Included in the table are the 15 substrates which consistently differed between strains in all 3 tests.

^b^The MSHR5848 variants were incubated in GENIII plates on the Omnilog®, and the data were analyzed by the Retrospect software. The respiratory activity, reported as arbitrary OmniLog units (OU), was normalized on a 0–100 scale (threshold values), for differences in metabolism for the chemicals. The threshold cut-off values for each response (“X”) were the following: X < 20 (negative, white), 20 < X < 80 (borderline, light grey), and X ≥ 80 (positive, dark grey)

^c^The Rough variant’s values were within 2 units of the cut-off (20) for the threshold negative group.

#### Biolog phenotype microarrays

**Fig 2:**
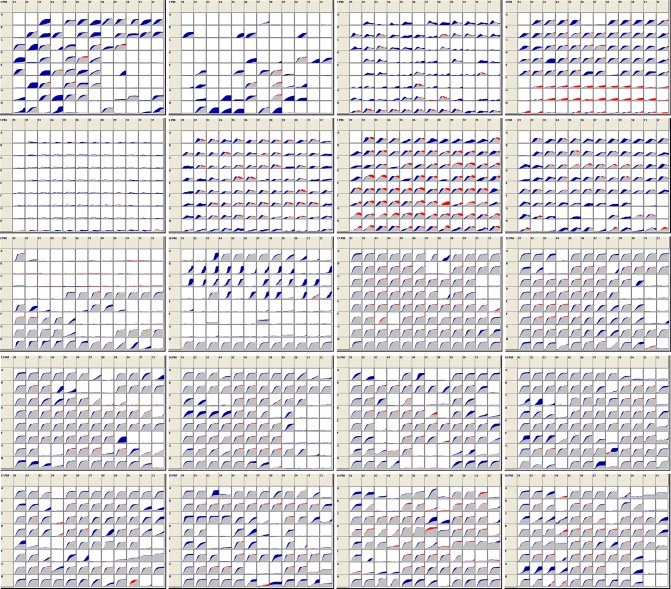
Phenotypic microarray data. The 20 PM plates are used to identify strain differences in ability to metabolize different sources of carbon, nitrogen, phosphorus or sulfur; or differences in sensitivity to antimicrobial conditions. Kinetic data were collected every 15 min for 48 h and the variant curves were compared, as shown. **Gray**: overlapping activity; **blue** area: greater Smooth response; and **red** area: greater Rough activity.

The Biolog Phenotype Microarray^TM^ system employs 20 microplates with a total of 1,920 substrates. This system is used to characterize isolates and identify strain differences in their ability to use different compounds as sources of carbon, nitrogen and phosphorus or sulfur; or in their sensitivity to stressful environmental conditions such as pH extremes or high salt concentrations; and in their sensitivity to antimicrobial chemicals such as antibiotics, detergents, and oxidizing agents. The data generated from these plates was consistent with the findings from the GEN III experiments. Table 4 lists the substrates producing the greatest differences in response between the Smooth and Rough variants. More detailed lists of the substrates producing significant differences in response between the variants are provided in S4 Table. Additionally, Fig 2 shows a snapshot of the 48 h kinetic data across all 20 plates. The Smooth variant is represented in blue while the Rough variant is represented by red. The gray portion of the curve represents the overlap, where the response was the same between the two variants. As shown by the greater number of blue curves, the Smooth variant was generally more metabolically active than the Rough variant and produced greater responses overall across all 20 plates compared to the Rough variant. The greatest differences were observed in metabolic pathway assays (plates 1–4 in Fig 2), specifically in the carbon source plates, though also in resistance to certain stress conditions, e.g., low pH and toxic ions (Table 4A). It should be noted that although the variants differed in growth rate, as described above, the PM platform measures cellular metabolism (respiration) and not the increase in cell numbers and biomass. When a strain stops multiplying or even fails to grow, it may continue to metabolize the substrate. To further examine the association between growth rate and respiration activity, PM plates were inoculated and incubated with Smooth and Rough variants and absorbance readings were measured for comparison to the previous PM results. The Smooth and Rough turbidity data agreed with the relative metabolic activities for the carbon, sulfur, and dipeptide nitrogen substrates shown in Table 4, suggesting that the differential metabolism by the variants was not significantly impacted by growth rate differences.

**Table 4:** Differences in responses of MSHR5848 variants to PM substrates.

**A: Smooth**			
PM Plate	No. wells	Substrate	Class or Mode of Action
1	6	L-Threonine	C-Source
		Citric Acid	C-Source
		L-Asparagine	C-Source
		D-Glucose-1-Phosphate	C-Source
		D-Galactonic Acid-g-Lactone	C-Source
		N-Acetyl-D-Glucosamine	C-Source
2	3	L-Pyroglutamic Acid	C-Source
		Putrescine	C-Source
		D,L Carnitine	C-Source
10	2	pH 4.5	pH, growth at 4.5
		pH 4.5 + L-Methionine	pH, decarboxylase
13	1	Thallium (I) acetate	toxic cation
14	1	Cadmium Chloride	transport, toxic cation
15	1	Guanidine hydrochloride	membrane, chaotropic agent
16	3	Chloroxylenol	Fungicide, disinfectant
		Dichlofluanid	fungicide, phenylsulphamide
		Potassium Tellurite	transport, toxic anion
17	1	Aminotriazole	histidine biosynthesis, catalase
18	3	Ketoprofen	anti-capsule
		2-Phenylphenol	DNA intercalator
		Tinidazole	Mutagen, nitroimidazole
19	2	Umbelliferone	DNA intercalator
		Thioglycerol	reducing agent, thiol, adenosyl methionine antagonist
20	2	Captan	fungicide, carbamate, multisite
		8-Hydroxyquinoline	chelator, lipophilic
**B: Rough**			
PM Plate	No. wells	Substrate	Class or Mode of Action
4	15	L-Methionine	S-Source
		N-acetyl-D,L Methionine	S-Source
		L-Cysteine	S-Source
		D-Methionine	S-Source
		L-Methionine Sulfoxide	S-Source
		L-Cysteinyl-Glycine	S-Source
		Glycyl-L-Methionine	S-Source
		Glutathione	S-Source
		Cystathionine	S-Source
		Lanthionine	S-Source
		D-Cysteine	S-Source
		Thiophosphate	S-Source
		Dithiophosphate	S-Source
		L-Djenkolic Acid	S-Source
		D,L-Ethionine	S-Source
7	5	tryptophan-Asp	N-source, dipeptide
		tryptophan-tyrosine	N-source, dipeptide
		tryptophan-phenylalanine	N-source, dipeptide
		phenylalanine-tryptophan	N-source, dipeptide
		phenylalanine-Serine	N-source, dipeptide
15	1	Domiphen bromide	membrane, detergent, cationic, fungicide
17	1	Phenylarsine Oxide	tyrosine phosphatase
19	3	Iodonitro Tetrazolium Violet	respiration
		Coumarin	DNA intercalator
		Harmane	imidazoline binding sites, agonist

While the responses of Rough overall were relatively low, this variant clearly outperformed the Smooth variant in the presence of sulfur sources and aromatic amino acid-containing nitrogen sources (plates 4, 6, and 7 in Fig 2, Table 4B, and S4 Table). Interestingly, none of the substrates in plate 5 (nutrient supplements) supported metabolism in either variant. Though less apparent than the metabolic substrate differences, the Smooth variant generally outperformed the Rough variant in the chemical sensitivity assays (plates 11–20 in Fig 2, Table 4, and S4 Table) with possible greater resistance to certain inhibitory conditions such as pH and antimicrobial substrates including potassium tellurite (reactive oxygen inducer); membrane-, DNA-, and chemically-active substances; and toxic charged molecules and microbicides. Thus these data suggest that the Smooth variant is more adaptable to a broader range of metabolic input but in the presence of inhibitors or stressors, the two variants often behave similarly.

### Phenotyping of LPS O polysaccharides

**Fig 3:**
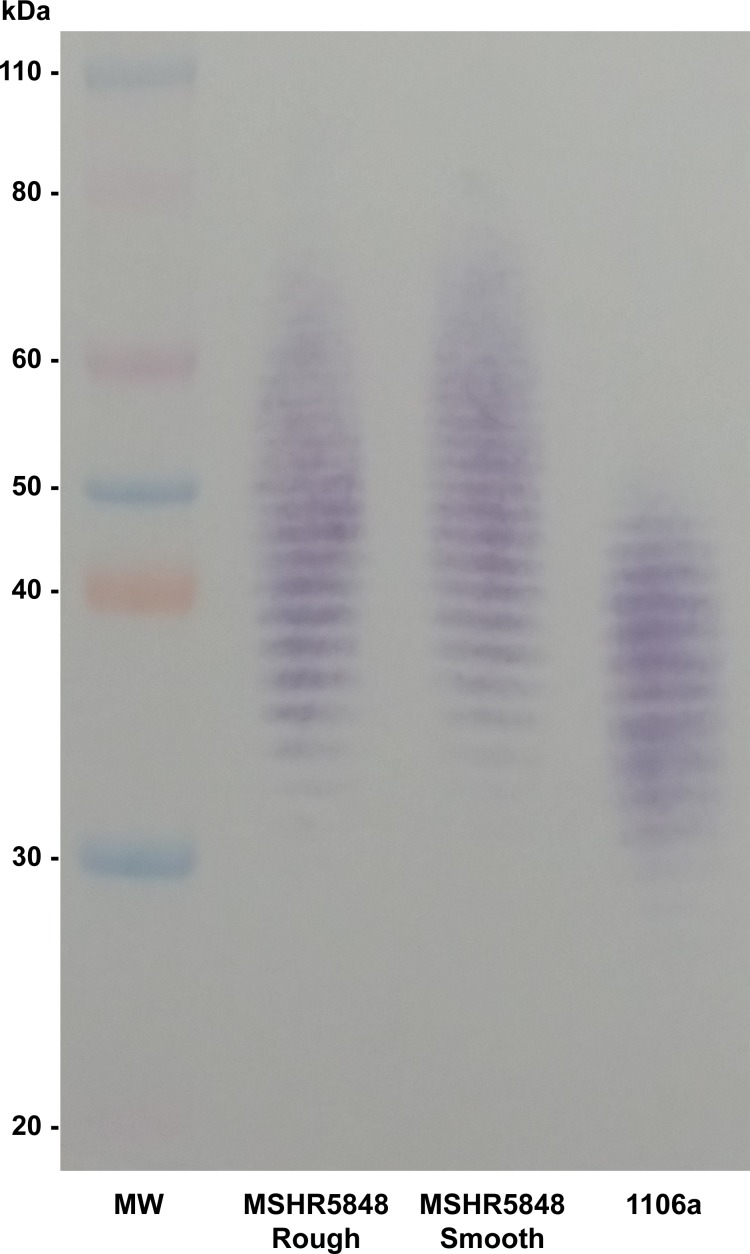
Western blot of LPS from *Bp* strains. Purified LPS from MSHR5848 Smooth and Rough, and *Bp* strain 1106a, were separated by SDS-PAGE and a western blot was done using monoclonal antibody (mAb) 11G3-1, specific for *Bp* LPS O polysaccharide (OPS). Unlike the typical Type A banding pattern of most *Bp* strains (1106a), the variants displayed identical higher molecular weight patterns.

Purified LPS samples from the Smooth and Rough variants of MSHR5848 were separated by SDS-PAGE and probed with monoclonal antibody 11G3-1, which is specific for *Bp* LPS. Smooth and Rough appeared to be identical in their LPS O polysaccharide (OPS) banding, with a range higher than that of the typical type A LPS banding pattern, as illustrated for *Bp* strain 1106a (Fig 3). We previously described three subtypes for *Bp* type A LPS based on banding pattern range [8], and the LPS of the two MSHR5848 variants can be classified as A3, which signifies the highest range for the subtypes. This A3 banding pattern was also observed for the two variants using a variety of other monoclonal antibodies specific for *Burkholderia* LPS, as well as by silver staining (data not shown). Interestingly, monoclonal antibody 9D5 was unable to recognize LPS from either Smooth or Rough, suggesting that both of these phenotypic variants have OPS structures that differ from typical type A strains. This antibody is specific for a unique conformational epitope found on OPS from typical type A *Bp* strains, and the absence of binding indicates alteration of the OPS acetyl and methyl substitution pattern for both variants [22].

### Persister phenotype

As demonstrated for various bacteria, the *Burkholderia* are thought to produce persister cells which may be a reservoir for chronic infections. These phenotypically altered forms can remain viable in the presence of adverse conditions such those present in the macrophage phagolysosome. In assays performed as described previously [41], both the Smooth and Rough variants appeared to produce antibiotic tolerant cells to a comparable extent in cultures treated with ciprofloxacin after they entered early stationary phase but not in those initially exposed to the antibiotic at early log stage (S1 Fig). These data suggested that persister cell formation, as generated in this model system, might not be a phenotype controlled by the variant switching mechanism in MSHR5848.

### Molecular genetic analysis

Comparisons of the restriction digest patterns for the Smooth and Rough variants showed that the ribosomal RNA of MSHR5848 was conserved in the variants (data not shown). Similarly, the sequence type (ST) using the *Bp* MLST scheme [51] derived from seven housekeeping genes was 553 for both variants (data not shown). This ST is consistent with that obtained elsewhere for the strain MSHR5848, according to the mlst.net database.

**Fig 4:**
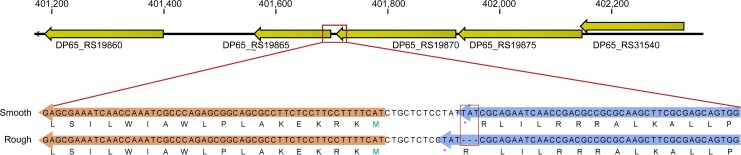
Comparison of the MSHR5848 variants to identify WGS differences. Consensus sequences for Smooth and Rough were aligned to identify significant sequence variations. A three base sequence (TAT) located on MSHR5848 chromosome 2 positions 401,708–401,710, was present in the Smooth but not in the Rough WGS (orange box). The indel is within the presumed promotor region of a gene annotated as a putative lipoprotein (DP65_RS19865/DP65_3853) and nearly identical to a hypothetical protein encoded on the genome of *Burkholderia* bacteriophage phiE12-2.

Using high-throughput, single-molecule sequencing (PacBio) we assembled complete genomes from DNA extracted from broth cultures grown from single colonies of Smooth and Rough. The two assemblies were nearly identical in length (chromosome 1: 4,054,192–4,054,259 bp; chromosome 2: 3,236,690 bp) and exhibited perfect synteny (i.e., no large-scale rearrangements or deletions) with each other and with the MSHR5848 reference assembly (chromosome 1: 4,054,219 bp; chromosome 2: 3,236,215 bp) [56]. Analysis of deep sequencing data (Illumina) from the broth cultures and the colony picks identified only a single genetic difference that was consistent between the two types: the presence of three bases in Smooth that were absent in Rough (an indel). These bases were missing in all of the reads obtained from the Rough broth cultures and colony picks (243x and 66x sequencing depth, respectively); but they were present at 94% frequency in the Smooth broth culture (181x) and in all of the reads from the Smooth colony picks (37x). This difference was also confirmed via PCR amplification and Sanger sequencing of five independently derived single colony stocks, each of Smooth and Rough morphotype. The three differentiating bases, TAT, correspond to MSHR5848 chromosome 2 positions 401,708–401,710, as illustrated in Fig 4. This indel occurs at the 3’ end of the predicted open reading frame for a hypothetical protein, DP65_RS19870 (GenBank: WP_038760828.1), but does not result in any changes to the predicted length or amino acid sequence for this protein. Notably, this indel is only 11 bp upstream from, and therefore potentially within a promoter region for, a protein annotated as a putative lipoprotein [DP65_RS19865/DP65_3853 (previously D65_3853), GenBank: WP_038760831.1] and exhibits 96% nucleotide identity to a hypothetical protein encoded on the genome of *Burkholderia* phage phiE12-2 (Genbank accession CP000624).

### Infection of macrophages and mice

**Fig 5:**
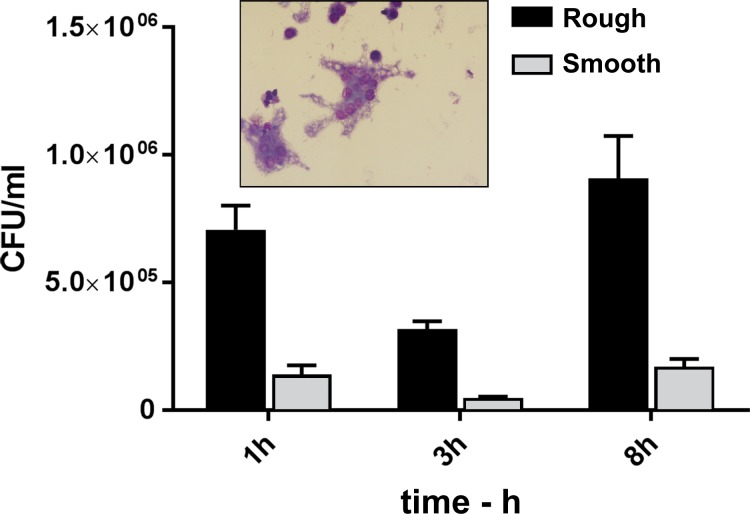
Infection of J774.A1 macrophage cultures with the Smooth and Rough variants of *Bp* MSHR5848. The MOIs were 19.4 and 19.1, respectively. The MSHR5848 Smooth was phagocytosed to an almost fivefold greater extent than the MSHR5848 Rough strain, as shown by the 3 h viable counts. The counts recovered from Smooth-infected cells at all three time point were greater than those from Rough-infected macrophages (p < 0.0001).

The ability of the Smooth and Rough variants to infect, survive in and induce cytotoxicity for J774.A1 macrophages was determined. Smooth was phagocytosed to an almost five-fold greater extent than Rough, as shown by the viable counts collected after the 1 h uptake and 2 h antibiotic treatment incubations (Fig 5). The viable counts recovered from Smooth-infected cells at all three time points, to include the final 8 h samples, were greater than those from Rough-infected macrophages (p < 0.0001). In addition, as shown in Table 5, the Smooth variant was more cytotoxic for the cells; Smooth induced more cell death and cell detachment/loss from wells than did Rough, and was associated with a greater extent of MNGC formation, as evidenced by the larger proportion of MNGCs and the greater mean number of nuclei within the MNGCs. The greater cytotoxicity and cell loss associated with Smooth suggests that the 8 h viable counts likely underestimated the extent of the intracellular replication by Smooth.

**Table 5:** Cytotoxicity of the Smooth and Rough variants of *Bp* MSHR5848 in J774.A1 macrophage cultures.

			MNGC or necrotic	MNGC
Variant	% cell loss	% cells dead (TB)^a^	cells (% of total)^b^	nuclei (%)^b^
Smooth	75	75	17.7	67.2
Rough	15	12.5	3.6	14.5

^a^TB: determined by trypan blue staining and does not include unstained necrotic (dead) cells.

^b^The extensive cell loss induced by the Smooth infection minimized the significance of these values.

**Fig 6:**
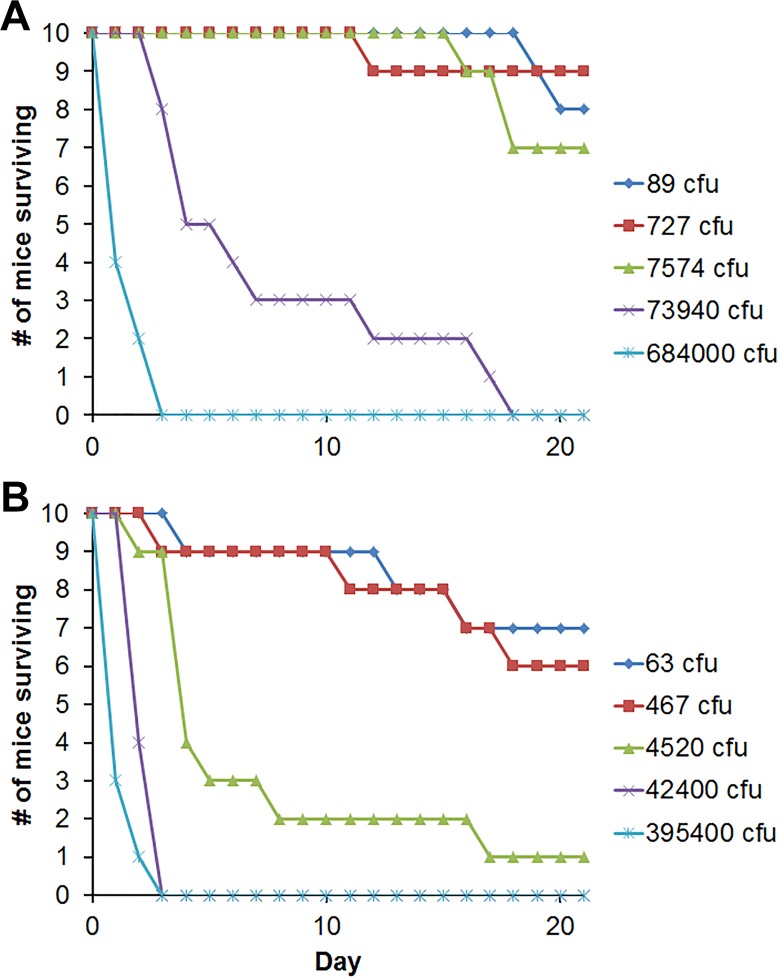
Comparison of the virulence of variants Smooth and Rough in a mouse infection model. BALB/c mice were challenged IP with five ten-fold dilutions of Smooth (panel A) or Rough (panel B), as described previously [11]. The decline in numbers of mice surviving with time for each dose group (10 mice/group) is shown. Infection with Rough was associated with more lethality/morbidity and a faster loss of survival than infection with Smooth.

The virulence of the variants was compared using BALB/c mice challenged by the IP route, a model useful for *Burkholderia* strain comparisons [8]. Both the day 21 and day 60 LD_50_ values for the Rough variant were about tenfold less than those of the Smooth variant, and the day 21 values were significantly different with a probability ≥ 95% by Bayesian probit analysis (Table 6). The greater decline in survival of mice infected with Rough compared to Smooth is illustrated in Fig 6. Also, at day 21, Rough exhibited greater potency at all doses associated with lethal rates ranging from 23% to 92% mortality, with a probability of > 95%, as shown in S2 Fig. Although the lower day 60 LD_50_ of Rough was not significantly different from that of Smooth, Rough exhibited significantly greater potency at all doses associated with lethal rates ranging from 79% to 93% mortality with a probability of > 95% (data not shown).

**Table 6:** Comparison of relative virulence for mice of strains derived from *Bp* MSHR5848.

	Day 21^a^			Day 60^a^		
		95% Credible Interval			95% Credible Interval	
Variant	Dose, CFU	Lower	Upper	Dose, CFU	Lower	Upper
Smooth	5.18 x 10^3^	1.67 x 10^3^	1.7 x 10^4^	1.12 x 10^2^	3.58	8.98 x 10^2^
Rough	4.69 x 10^2^^b^	1.38 x 10^2^	1.42 x 10^3^	15.1^b^	0.01	1.51 x 10^2^

^a^BALB/c mice were challenged by the IP route with the Smooth or Rough variant of *B*. *pseudomallei* MSHR5848, and the mice were monitored for morbidity and mortality for 60 days. The day 21 and 60 survival data were evaluated statistically to determine LD_50_ values and compare virulence potencies by using Bayesian probit analysis as described in the methods.

^b^Both the day 21 and day 60 LD_50_ values of Rough were approximately tenfold less than that of Smooth; and the day 21 LD_50_ values were significantly different with a probability ≥ 95% by Bayesian probit analysis.

## Discussion

The initial objective of this study was to phenotypically and genotypically characterize the major variants of MSHR5848. Our major goal is to determine the mechanism of variant expression and the role of this phenomenon in disease pathogenesis. We hypothesized that the variants may provide a model for identifying *in vitro* functions associated with different stages of an evolving infection.

The MSHR5848 Smooth and Rough variants differed in numerous phenotypes to include colony/cell morphology, biochemical sensitivity or utilization, macrophage survival and activity, and animal virulence. Both variants were present in the source vial although in different proportions yet both were relatively stable when passaged individually under routine laboratory conditions for the preparation of single colony isolated stocks. Nevertheless, both Smooth and Rough were capable of reverting to the alternate morphotype under certain conditions, albeit in a stochastic manner. The Rough variant reverted more frequently overall and after exposure to a wider range of stressful *in vitro* conditions than did the Smooth variant. Reversion of the latter was only observed with Smooth variants freshly isolated from a Rough single colony stock incubated under one condition. This disparity in the extent and randomness of variant switching is a common finding reported previously for *Bp*, as described below.

The production of distinct morphological variants by strains of *Bp* is well-established, and the activity of MSHR5848 appears to be a modification of this phenomenon. For example, the rough variant described by Nicholls [23] was present in much greater proportion than the mucoid variant, in agreement with the greater proportion of Rough to Smooth variants in the MSHR5848 source vial and seed stocks. However, we observed greater stability and a lower incidence of switching than reported previously [23] for both Smooth and Rough grown under typical laboratory conditions.

The rough morphotype I described by Chantratita and colleagues [12] appeared to be the parent from which the others originated *in vivo* and *in vitro* in response to stressful conditions. Despite strain heterogeneity, evidence from the previous work and our studies with MSHR5848 support the idea that different colony morphotypes may reflect adaptive changes that enhance fitness in a particular environment [12, 13]. As shown previously, [21], *Bp* strain K96243 produced colony variants which differed in morphology and in their extent of growth in low oxygen or acidic environments, despite being genetically identical. The variants of both the K96243 and MSHR5848 strains were relatively stable but could switch morphotypes at a stochastic rate, exhibited variant-dependent production of extracellular DNA, and survived differentially *in vivo* [21]. Only one of the K96243 types, albeit attenuated, could persist in the harsh conditions of the murine stomach and colonize the mucosa. Similarly, MSHR5848 Smooth was less virulent than Rough for mice but Smooth exhibited greater resistance to adverse conditions, e.g., the macrophage environment and inhibitory chemicals. Thus survival in a defined niche such as the gastric mucosa or splenic macrophages may not necessarily be predictive of animal survival. Wikraiphat et al. described mucoid (M) and nonmucoid (NM) variants of clinical isolates which exhibited antigenically different OPS [22]. Switching from NM to M occurred for more pairs and under a larger variety of *in vitro* growth conditions than the reverse, similar to the findings with MSHR5848 variants. Furthermore, a greater extent of phagocytosis by macrophages was associated with M compared to NM, again similar to the MSHR5848 variants. Finally, Gierok and coworkers isolated several colony morphotypes from two *Bp* strains after their extended growth under nutrient starvation conditions. The morphotypes varied metabolically in nutrient uptake and secretion and in recovery from infected macrophage cultures and from the lungs of infected mice. Interestingly, for at least one of the strains, passage of the variants in macrophages and in mice selected for isolates all having the same morphotype and similar metabolic profiles [14]. This infection-induced reduction in variant types supports the role of environmental stressors in conversion to a maximally fit variant(s) [9, 12–14, 21].

There are several possibilities which could potentially explain the basis for the Smooth, in contrast to Rough, colony morphologies of MSHR5848. A role for OPS modifications, as described previously [26], is unlikely since Smooth and Rough produce antigenically identical OPS and antibody 9D5 was unable to recognize OPS from either variant [22]. Secondly, a role for the *Bp* polysaccharide capsule (CPS) is possible. The CPS expressed by human *Bp* isolates is surface-associated, a proven virulence factor, and induces protective anti-CPS antibodies [57–62]. Thirdly, as will be discussed, the mechanism driving MSHR5848 colony morphology could be similar to that which influences the strain K96243 variant phenotypes described previously [21]. The MSHR5848 variant differences in LPS, CPS, and DNA secretion are being further characterized in efforts to better understand the basis of their distinct colony morphologies.

Although the phenotype reversion of MSHR5848 appeared to generally resemble the behavior described previously for other *Bp* strains, it was accompanied by alterations in a more diverse array of phenotypes than reported previously [19, [14, 21, 22]. The broad and pleiotropic nature of the phenotypes impacted by MSHR5848 variant switching suggests a role for a master regulator with global control over multiple genes. The complex nature of the *in vivo* niches for *Bp* poses challenges in efforts to interpret *in vivo* significances of the *in vitro* responses. Nevertheless, metabolic responses appear to be involved in pathogen niche adaptation and virulence [24, 38, 63, 64]. To exemplify this concept, the ability of the Smooth variant to utilize a greater diversity of carbon sources may lead to an initial growth advantage when exposed to nutrient-rich niches, such as the infected lung. For instance, the Smooth variant preferentially used the compounds putrescine and carnitine. Such polyamines are widely distributed, are essential for normal growth of prokaryotic and eukaryotic cells, and play important roles in protection from osmotic and temperature extremes [65–67]. Such activity might also contribute to the greater resistance of Smooth to inhibitory substrates, as discussed below.

Despite a possible initial growth advantage of the Smooth variant in nutrient-rich niches, bacterial adaptation and deregulation of redundant carbon source utilization could lead to loss of metabolic pathways not required for growth in exchange for the expression of virulence factors required for enhanced persistence and virulence [9, 26, 34]. Interestingly, the less metabolically active Rough (as shown in Fig 2 and S4 Table) was more virulent than Smooth.

The Rough variant clearly outperformed Smooth in sole dipeptide nitrogen and sulfur source utilization. The capacity to import and utilize small peptides is common in many bacteria [68], in which they can serve as nitrogen and carbon sources in nutrient-poor environments. The host cell is limiting for free amino acids but has other molecules which can be degraded, producing oligopeptides as amino acid sources [38, 63, 69–75]. Since Rough preferentially utilized 16 dipeptides, it might be especially efficient in their uptake or use [76, 77]. The Rough variant was especially active in its respiration of dipeptide substrates containing aromatic amino acids, e.g., tryptophan, phenylalanine, and/or tyrosine. These molecules play significant roles in the biosynthesis and activity of microbial siderophores, which are essential for bacterial survival in the iron-limiting conditions in the host [2, 38] [78]. The pathogenic *Burkholderia* have multiple siderophore and hemin/hemoglobin uptake and utilization systems [79–84]. The more efficient mobilization of such systems by Rough might allow it to better utilize aromatic amino acid–containing substrates to promote its survival.

Sulfur and sulfur-containing compounds are essential for *in vivo* growth and persistence of *Bp*, but they and other essential nutrients vary greatly in availability depending on the niche and inflammatory response-induced adverse conditions such as hypoxia and acidity [13, 62, 63, 85–88]. The retrieval of amino acids scavenged from host-degraded protein is a critical pathogen adaptive response. This is especially important for amino acids which are essential but relatively scarce, such as cysteine [63]. Thus, the enhanced capacity of Rough to utilize a variety of peptide and cysteine sources such as glutathione (GSH) and related thiols (Table 4B and S4 Table) could promote its survival in amino acid and sulfur limiting environments. Wong et al. showed that intracellular *Bp* can sense GSH through its type six secretion system (T6SS) membrane histidine sensor kinase VirA as a signal to upregulate virulence genes [89]. After *Bp* escape from the phagolysosome, the T6SS is activated and mediates cell fusion, intercellular spreading and MNGC formation. The conversion of methionine to cysteine is a key early step in thiol synthesis in host cells. This finding suggests that Rough might be able to facilitate this virulence-promoting process. Finally, the high concentrations of N- and O-reactive species often generated in host oxidative responses to infection can induce changes such as the oxidation of thiosulfate to tetrathionate [24]. Tetrathionate inhibits growth of many Gram-negative bacteria, although certain pathogens can use tetrathionate as an energy source [24, 63]. Similarly, Rough was more metabolically active than Smooth for tetrathionate and thiosulfate substrates (S4 Table). However, these thiols might conversely represent stressors which can induce Rough to revert to the less responsive Smooth. In circumstances where various S sources are available, Smooth might thus compete better than Rough for the available carbon (Table 4A and S4 Table) [90, 91].

To examine the potential roles these substrates have in morphotype reversion, preliminary studies were done to assess the extent to which growth in the presence of selected PM substrates promoted variant phenotypic switching. Rough was capable of reverting to Smooth in the presence of thiols as the sole sulfur sources (cystathionine, lanthionine, and glutathione), and Smooth reversion to Rough was rare and only observed in one incubation with cystathionine. Thus, both variants were capable of reversion *in vitro*, and it suggested that exposure to environments limited in specific nutrients might stimulate variant switching *in vivo*.

The responses of the variants to the antimicrobial compounds and conditions tested in PM plates 9–20 suggested an overall more extensive resistance of Smooth compared to Rough. A total of 50 of the 59 substrates that Smooth preferentially metabolized or resisted were inhibitory compounds or conditions. In contrast, only 13 of the 47 compounds to which Rough had a stronger response than Smooth were inhibitors. Hence, Smooth was more metabolically active under low pH conditions than Rough, resembling the greater reported persistence of the K96243 yellow variant (compared to the white) in the acidic conditions of the murine gastric mucosa [21]. Although Smooth was reduced in systemic virulence compared to Rough (resembling the K96243 variants), the greater resistance of Smooth to toxic host conditions may partially explain its greater infectivity and cytotoxicity in a macrophage model. *Bp* often localizes to the host macrophage where it must be able to resist the antimicrobial substances (e.g., AMPs and free radicals) which comprise the phagolysosomal defenses [13, 62, 85–88].

The inhibitor potassium tellurite is toxic to bacteria due to the generation of superoxide radicals and oxidative stress [92, 93]. Maintenance of the critical cell oxidation-reduction balance is achieved by reduced environment sulfur sources, especially GSH [93–95]. Little is known about the mechanisms of resistance to tellurite [92], but Smooth may express a mechanism conferring greater resistance to tellurite compared to Rough. Smooth also exhibited greater metabolic activity in the presence of various other antimicrobial cations, anions, mutagens, chelators, and reducing agents which may model exposure to adverse host environments (Table 4A). For example, thallium acetate and cadmium chloride are toxic cations which suppress growth of many organisms, and hydroxyquinolone is a strong chelator for metals such as iron and metals necessary for the catalysis of DNA biosynthesis [96, 97]. The compound chloroxylenol has disinfectant activity which elicited a more resistant response by Smooth compared to Rough. In studies to assess variant reversion, Smooth initiated growth but was susceptible to chloroxylenol-mediated killing and showed a variable but high degree of reversion (50%) to Rough. Although the Rough cells were inhibited in growth, revertants were not observed and the inoculum remained viable. Thus, perhaps the stress imposed by the chemical induced reversion of Smooth to Rough at a high rate. Studies are needed to identify the basis of resistance to these inhibitors and the role of *Bp* metabolic and resistance differences in pathogenesis.

The collective substrate utilization and antimicrobial sensitivity data therefore suggests that Smooth might grow initially to a greater extent than Rough in harsh environments such as the host phagolysosome. Nevertheless, residence in the phagolysosome is a transient phase in phagocyte infection for *Bp*, which can then escape to the cytoplasm where they mobilize actin, spread to other cells, and facilitate MNGC formation. Such functions are perhaps better carried out by Rough. Although *in vitro* responses cannot be directly translated to *in vivo* growth or virulence, the examples described here support the concept that pathogens adapt metabolically to environmental niches which differ in available metabolic substrates and in antibacterial activitiy in a manner allowing them to evolve during infection. The interactions might result in either chronic persistence or increased virulence of the bacteria [24, 26, 27, 34, 38].

The cell culture and murine infection models provided further evidence that phenotypic variation of MSHR5848 may impact infection. In contrast to the reduced virulence of Smooth compared to Rough for mice, Smooth replicated to a greater extent and was more cytotoxic for macrophages than Rough. These observations agree with the inverse relationship between *in vitro* macrophage and *in vivo* virulence observed for several *Bp* strains [8].

The molecular mechanism of MSHR5848 phenotypic variation is unknown, but the broad and reversible nature of the phenotypes impacted by switching suggests the involvement of a master regulatory locus or loci with global control over numerous genes. Whole genome comparisons of Smooth and Rough confirmed that they are genetically conserved, and sequencing revealed only one consistent genetic difference between Smooth and Rough: three bases that are present in Smooth and absent in Rough. This indel potentially disrupts the promoter region for a gene annotated as a hypothetical, potentially transmembrane, lipoprotein (software tools Ensembl Bacteria release 23-EMBL-EBI and TMHMM) on MSHR5848 chromosome 2. Furthermore, this protein sequence was found to be nearly identical to that of an ORF (hypothetical protein gp19) in the complete phage genome of *Burkholderia* phage phiE12-2 [98]. However, the original source and significance of this finding are unknown. To further clarify the results, transcriptomic and mutational analyses are being pursued to evaluate the potential significance of variant sequence differences on the phenotypic diversity of MSHR5848.

A transcriptional regulator controlling colony color variation (*yelR*) was identified in the studies of Austin et al. [21]; however, it is not known whether this gene or other mechanism controls expression of the other phenotypes described for K96243. It was hypothesized that a master regulator, in the form of a bistable switch, may be involved in the regulation of the K96243 phenotypes, as described by Dubnau et al. [21, 99]. Although the mechanism(s) responsible for *Bp* phenotypic diversity are undefined, the production of variants with multiple reversible phenotypes in the presence of environmental stressors suggests that post-transcriptional or -translational events are the immediate inducers of the reverting phenotypes described previously for *Bp* [12, 14, 20, 22, 35]. As a result, additional research on the basis and importance of *Burkholderia* phenotypic plasticity is clearly warranted.

## Supporting information

S1 Fig Persister phenotype of MSHR5848. (TIF)

S2 Fig Comparison of Smooth and Rough virulence in mice. (TIF)

S1 Table MSHR5848 colony morphologies in original, seed and production stocks: Summary. (DOCX)

S2 Table Variants derived from BURK178 seed and production stock morphotypes: Summary. (DOCX)

S3 Table Responses of MSHR5848 variants to selected MIS fatty acid peaks. (DOCX)

S4 Table PM substrates by difference value. (XLSX)
