# Correction: Two stable variants of *Burkholderia pseudomallei* strain MSHR5848 express broadly divergent *in vitro* phenotypes associated with their virulence differences

**DOI:** 10.1371/journal.pone.0215200

**Published:** 2019-04-04

**Authors:** A. A. Shea, R. C. Bernhards, C. K. Cote, C. J. Chase, J. W. Koehler, C. P. Klimko, J. T. Ladner, D. A. Rozak, M. J. Wolcott, D. P. Fetterer, S. J. Kern, G. I. Koroleva, S. P. Lovett, G. F. Palacios, R. G. Toothman, J. A. Bozue, P. L. Worsham, S. L. Welkos

In Fig 5, there is a labeling error. The names in the figure legend are reversed. The black bar should say “Smooth” and the gray bar should say “Rough.” Please see the complete, correct Fig 5 here.

**Fig 5:**
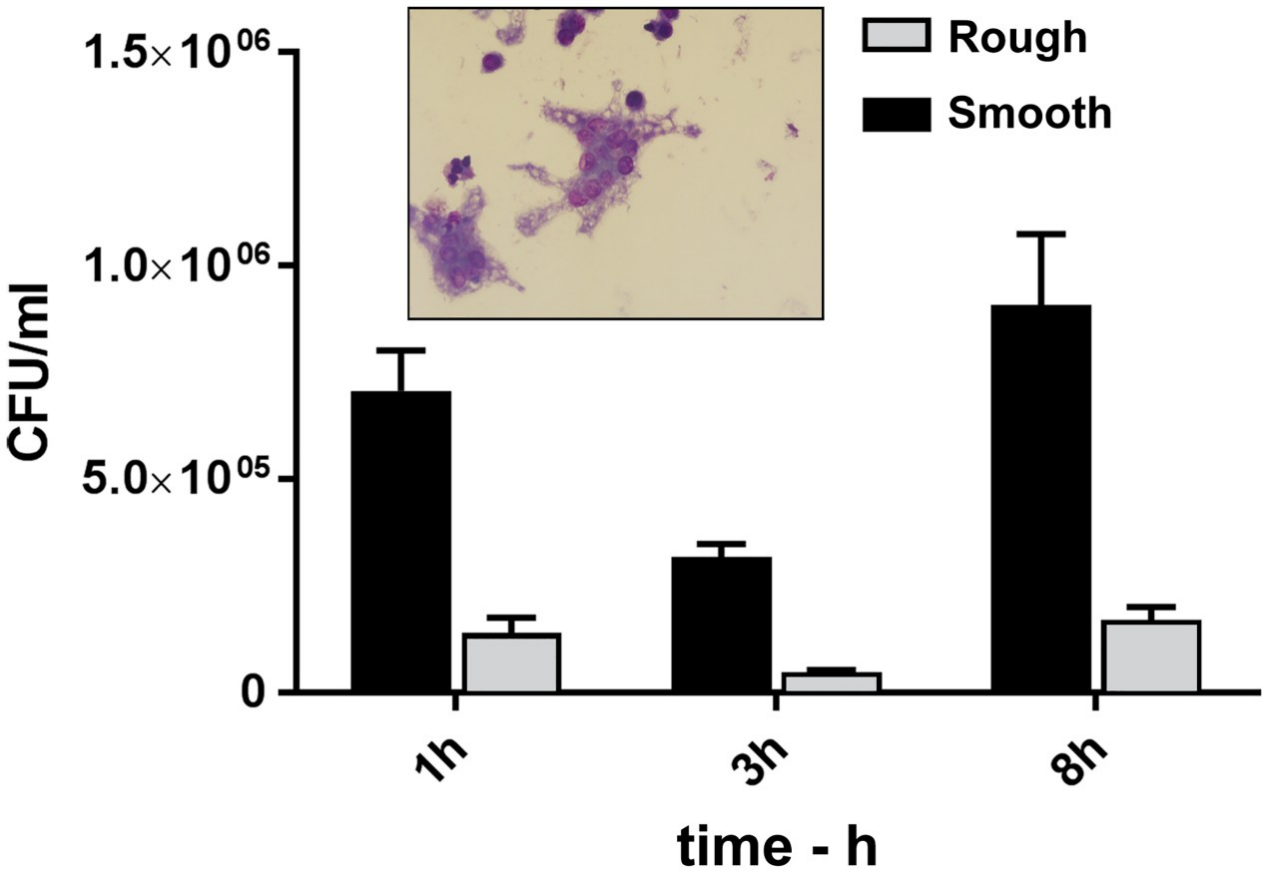
Infection of J774.A1 macrophage cultures with the Smooth and Rough variants of *Bp* MSHR5848. The MOIs were 19.4 and 19.1, respectively. The MSHR5848 Smooth was phagocytosed to an almost fivefold greater extent than the MSHR5848 Rough strain, as shown by the 3 h viable counts. The counts recovered from Smooth-infected cells at all three time point were greater than those from Rough-infected macrophages (p < 0.0001).
