# Supplementary figures and images for: Two stable variants of Burkholderia pseudomallei strain MSHR5848 express broadly divergent in vitro phenotypes associated with their virulence differences
Source: PLoS One. 2017 Feb 10;12(2):e0171363. doi: 10.1371/journal.pone.0171363 (PMC5302386; doi:10.1371/journal.pone.0171363)

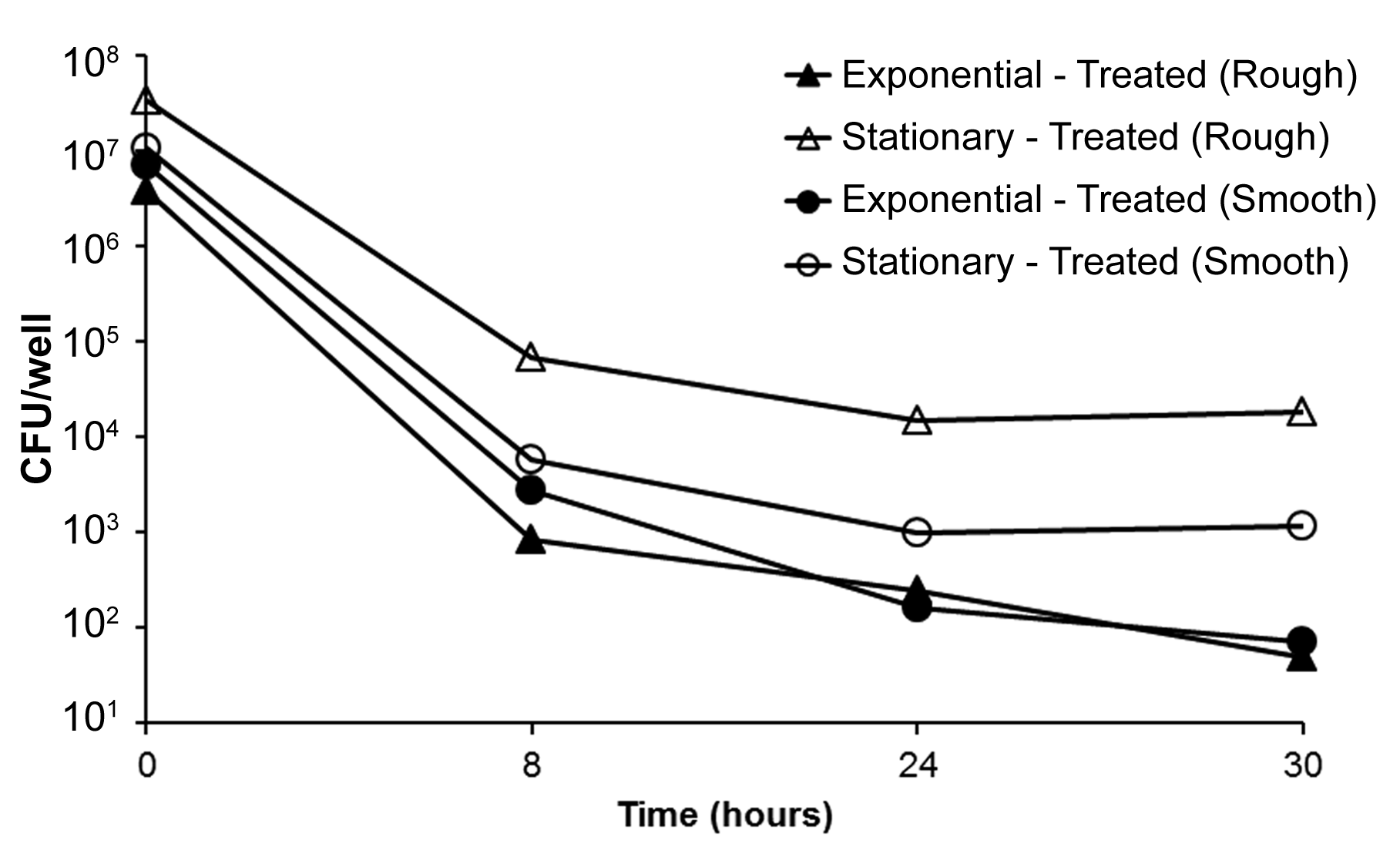

Supplement: S1 Fig — (TIF) [file pone.0171363.s001.tif]

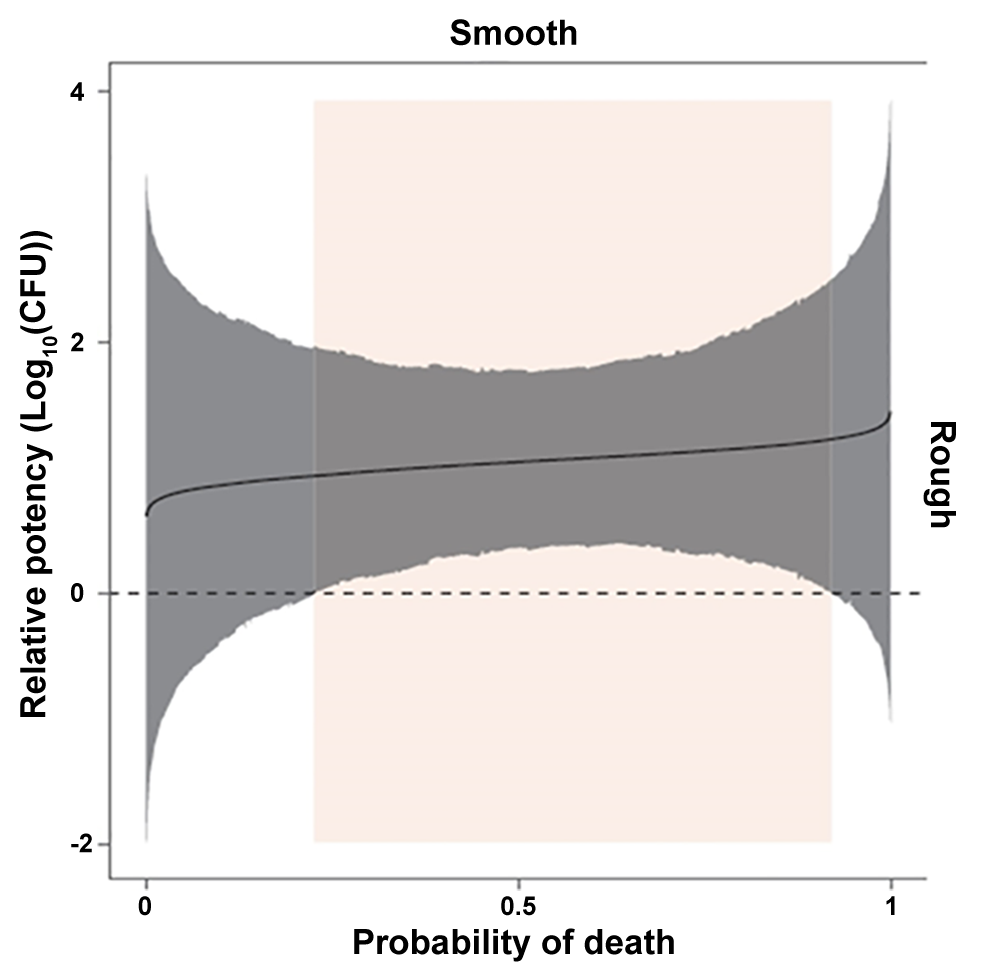

Supplement: S2 Fig — (TIF) [file pone.0171363.s002.tif]
