# Supplementary material for: Two stable variants of Burkholderia pseudomallei strain MSHR5848 express broadly divergent in vitro phenotypes associated with their virulence differences
Source: PLoS One. 2017 Feb 10;12(2):e0171363. doi: 10.1371/journal.pone.0171363 (PMC5302386; doi:10.1371/journal.pone.0171363)
Supplement: S1 Table — (DOCX) [file pone.0171363.s003.docx]

| **S1Table.** **MSHR5848 Colony morphologies in original, seed and production stocks: SUMMARY** | | | | | | | | | |
| --- | --- | --- | --- | --- | --- | --- | --- | --- | --- |
|  |  |  |  |  |  |  |  |  |  |
|  |  | Variants^a^ | | | | | |  |  |
|  |  | Smooth | | Rough | | Mucoid^b^ | | TOTAL |  |
| Stock | Dilution | No. CFU | % | No. CFU | % | No. CFU | % | No. CFU |  |
| Original | 10^-5^ | 0 | 0 | 67 | 100 | 0 |  | 67 |  |
|  | 10^-4^ | 3 | 0.38 | 776 | 99.6 | 0 |  | 779 |  |
|  | Total | 3 | **0.35** | 843 | **99.6** | 0 | 0 | 846 |  |
| Master seed^c^ | 10^-7^ | 5 | 4.3 | 111 | 95.7 | 0 |  | 116 |  |
|  | 10^-6^ | 36 | 4.75 | 720 | 95 | 2 |  | 758 |  |
|  | Total | 41 | **4.7** | 831 | **95.1** | 2 | 0.23 | 874 |  |
| Production stock^c^ | 10^-7^ | 37 | 48.7 | 37 | 48.7 | 2 |  | 76 |  |
|  | 10^-6^ | 289 | 46.7 | 315 | 50.9 | 15 |  | 619 |  |
|  | Total | 326 | **46.9** | 352 | **50.6** | 17 | 2.4 | 695 |  |
| ^a^Data shown are the total no. colonies on triplicate plates for each of two dilutions.  ^b^Unstable random variants of Smooth (mucoid with irregular or unformed, runny edge) | | | | | | |  |  |  |
| ^c^Stocks were made using solid media. | | | | | | | | |  |
