# Supplementary material for: Two stable variants of Burkholderia pseudomallei strain MSHR5848 express broadly divergent in vitro phenotypes associated with their virulence differences
Source: PLoS One. 2017 Feb 10;12(2):e0171363. doi: 10.1371/journal.pone.0171363 (PMC5302386; doi:10.1371/journal.pone.0171363)
Supplement: S2 Table — (DOCX) [file pone.0171363.s004.docx]

| **S2 Table**. Variants derived from BURK178 seed and production stock morphotypes - Summary | | | | | | | |
| --- | --- | --- | --- | --- | --- | --- | --- |
|  |  |  |  |  |  |  |  |
|  |  | Variants produced from colony types - No. CFU^a^ | | | |  |  |
|  |  | Types 1 and 2 | | Unstable variants^c^ | |  |  |
| Stock^b^ and Colony type | Dilution | Yellow (Smooth) (%) | Rough white (Rough) (%) | Mucoid (%)^b^ | Flat smooth gray (%)^b^ | Total No. |  |
| SS rough white (**Rough**) | 10-7 | 0 | 34 | 0 | 0 | 34 |  |
|  | 10-6 | 0 | 251 | 0 | 0 | 251 |  |
|  |  | 0 (0) | 285 (100) | 0 (0) | 0 (0) | 285 |  |
| SS Yellow (**Smooth**) | 10-7 | 33 | 0 | 5 | 0 | 38 |  |
|  | 10-6 | 413 | 0 | 21 | 0 | 434 |  |
|  | Total: | 446 (92.3) | 0 (0) | 26 (2.6) | 0 (0) | 472 |  |
| MSS Mucoid | 10-6 | 71 | 0 | 31 | 2 | 104 |  |
|  | 10-5 | 629 | 0 | 108 | 42 | 779 |  |
|  | Total: | 700 (79.3) | 0 (0) | 139 (15.7) | 44 (5.0) | 883 |  |
| PS rough white (**Rough**) | 10-7 | 0 | 38 | 0 | 0 | 38 |  |
|  | 10-6 | 0 | 266 | 0 | 0 | 266 |  |
|  | Total: | 0 (0) | 304 (100) | 0 (0) | 0 (0) | 0 |  |
| PS Yellow (**Smooth**) | 10-7 | 116 | 0 | 7 |  | 123 |  |
|  | 10-6 | 1056 | 0 | 50 |  | 1112 |  |
|  | Total: | 1172 (94.9) | 0 | 57 (1.1) | 6 (0.5) | 1235 |  |
| PS Mucoid | 10-7 | 102 | 0 | 9 | 0 | 111 |  |
|  | 10-6 | 833 | 0 | 82 | 14 | 929 |  |
|  | Total: | 935 (90.8) | 0 | 90 (8.7) | 14 (1.4) | 1030 |  |
| ^a^The stocks were cultured on SBAP for 3 days at 37C and an isolated colony of the indicated type | | | | | | |  |
| was suspended in PBS. The suspension was adjusted to OD_620_ = 1.0, serially diluted and the | | | | | | |  |
| dilutions spread on each of three plates. The values shown are the total no. colonies on the | | | | | | |  |
| triplicate plates of each dilution; the data are representative. | | | | |  |  |  |
| ^b^SS - seed stock. PS - production stock | | | |  |  |  |  |
| ^c^Unstable variants produced by yellow Smooth colonies: a smooth flat grey variant, and large | | | | | | |  |
| raised mucoid variants with irregular edge or unformed runny edge | | | | |  |  |  |
|  |  |  |  |  |  |  |  |
