# Supplementary material for: Two stable variants of Burkholderia pseudomallei strain MSHR5848 express broadly divergent in vitro phenotypes associated with their virulence differences
Source: PLoS One. 2017 Feb 10;12(2):e0171363. doi: 10.1371/journal.pone.0171363 (PMC5302386; doi:10.1371/journal.pone.0171363)
Supplement: S3 Table — (DOCX) [file pone.0171363.s005.docx]

| **S3 Table**. **Responses of MSHR5848 variants to selected MIS fatty acid peaks** | | | | | |  |  |  |  |
| --- | --- | --- | --- | --- | --- | --- | --- | --- | --- |
|  | Peaks^a^ | | | | | | | | |
| Variant | 15:1 w6c | 16:1 w7c/16:1 w6c | 17:1 w7c | 17:0 cyclo | 18:1 w7c | 18:1 w9c | 17:0 iso 3OH | 19:0 cyclo w8c | 19:1 w6w/w7c/19cy |
| Smooth | 0.16 | 8.72 | 0.16 | 6.84 | 31.16 | - | - | 5.28 | - |
| Rough | - | 2.39 | - | 15.43 | 8.19 | 0.39 | 0.24 | 19.06 | 0.19 |
| ^a^Values shown are the percentage of the peak in relation to the whole profile | | | | | | |  |  |  |
| peak present in Smooth but not Rough | | |  |  |  |  |  |  |  |
| peak present in Rough but not Smooth | | |  |  |  |  |  |  |  |
|  |  |  |  |  |  |  |  |  |  |
